# Safety profile of the RTS,S/AS01 malaria vaccine in infants and children: additional data from a phase III randomized controlled trial in sub-Saharan Africa

**DOI:** 10.1080/21645515.2019.1586040

**Published:** 2019-04-23

**Authors:** Yolanda Guerra Mendoza, Elodie Garric, Amanda Leach, Marc Lievens, Opokua Ofori-Anyinam, Jean-Yves Pirçon, Jens-Ulrich Stegmann, Pascale Vandoolaeghe, Lucas Otieno, Walter Otieno, Seth Owusu-Agyei, Jahit Sacarlal, Nahya Salim Masoud, Hermann Sorgho, Marcel Tanner, Halidou Tinto, Innocent Valea, Ali Takadir Mtoro, Patricia Njuguna, Martina Oneko, Godfrey Allan Otieno, Kephas Otieno, Samwel Gesase, Mary J Hamel, Irving Hoffman, Seyram Kaali, Portia Kamthunzi, Peter Kremsner, Miguel Lanaspa, Bertrand Lell, John Lusingu, Anangisye Malabeja, Pedro Aide, Pauline Akoo, Daniel Ansong, Kwaku Poku Asante, James A Berkley, Samuel Adjei, Tsiri Agbenyega, Selidji Todagbe Agnandji, Lode Schuerman

**Affiliations:** a GSK, Wavre, Belgium; b KEMRI-Walter Reed Project, Kombewa, Kenya; c Kintampo Health Research Center, Kintampo, Ghana; d Diseases Control Department, London School of Hygiene and Tropical Medicine, London, UK; e Centro de Investigação em Saúde de Manhiça, Manhiça, Mozambique; f Faculdade de Medicina, Universidade Eduardo Mondlane (UEM), Maputo, Mozambique; g Muhimbili University of Health and Allied Sciences (MUHAS), Dar es Salaam and Ifakara Health Institute, Bagamoyo, Tanzania; h Institut de Recherche en Science de la Santé, Nanoro, Burkina Faso; i Swiss Tropical and Public Health Institute, Basel, Switzerland; j Epidemiology and Medical Parasitology department, University of Basel, Basel, Switzerland; k Kenya Medical Research Institute-Wellcome Trust Research Programme, Centre for Geographic Medicine Research, Kilifi, Kenya; l Pwani University, Kilifi, Kenya; m University of Oxford, Oxford, UK; n Kenya Medical Research Institute, Centre for Global Health Research, Kisumu, Kenya; o National Institute for Medical Research, Korogwe, Tanzania; p Division of Parasitic Diseases and Malaria, Center for Global Health, Centers for Disease Control and Prevention, Atlanta, GA, USA; q University of North Carolina Project, Lilongwe, Malawi; r Centre de Recherches Médicales de Lambaréné, Lambaréné, Gabon and Institute of Tropical Medicine, University of Tübingen, Tübingen, Germany; s Barcelona Institute for Global Health (ISGlobal), Hospital Clínic-Universitat de Barcelona, Barcelona, Spain; t National Institute of Health, Ministry of Health, Maputo, Mozambique; u Kwame Nkrumah University of Science and Technology, Kumasi, Ghana

**Keywords:** (5–10): Malaria, RTS,S/AS01 vaccine, safety, meningitis, febrile convulsions, cerebral malaria

## Abstract

A phase III, double-blind, randomized, controlled trial (NCT00866619) in sub-Saharan Africa showed RTS,S/AS01 vaccine efficacy against malaria. We now present in-depth safety results from this study. 8922 children (enrolled at 5–17 months) and 6537 infants (enrolled at 6–12 weeks) were 1:1:1-randomized to receive 4 doses of RTS,S/AS01 (R3R) or non-malaria control vaccine (C3C), or 3 RTS,S/AS01 doses plus control (R3C). Aggregate safety data were reviewed by a multi-functional team. Severe malaria with Blantyre Coma Score ≤2 (cerebral malaria [CM]) and gender-specific mortality were assessed *post-hoc*. Serious adverse event (SAE) and fatal SAE incidences throughout the study were 24.2%–28.4% and 1.5%–2.5%, respectively across groups; 0.0%–0.3% of participants reported vaccination-related SAEs. The incidence of febrile convulsions in children was higher during the first 2–3 days post-vaccination with RTS,S/AS01 than with control vaccine, consistent with the time window of post-vaccination febrile reactions in this study (mostly the day after vaccination). A statistically significant numerical imbalance was observed for meningitis cases in children (R3R: 11, R3C: 10, C3C: 1) but not in infants. CM cases were more frequent in RTS,S/AS01-vaccinated children (R3R: 19, R3C: 24, C3C: 10) but not in infants. All-cause mortality was higher in RTS,S/AS01-vaccinated versus control girls (2.4% vs 1.3%, all ages) in our setting with low overall mortality. The observed meningitis and CM signals are considered likely chance findings, that – given their severity – warrant further evaluation in phase IV studies and WHO-led pilot implementation programs to establish the RTS,S/AS01 benefit-risk profile in real-life settings.

## Introduction

The widespread implementation of malaria prevention and control measures, such as the use of insecticide-treated nets, improved diagnosis, and artemisinin combination therapy, has led to considerable gains in the control of malaria.^1,2^ Nevertheless, malaria remains a major public health threat, especially in young children in sub-Saharan Africa (SSA). In 2017, an estimated 219 million cases of malaria occurred worldwide, resulting in 435,000 deaths, of which 93% occurred in Africa. About 61% of all malaria deaths, mostly caused by *Plasmodium falciparum*, were estimated to occur in children younger than 5 years.^3,4^

The development and deployment of an effective malaria vaccine is considered a further important step towards reducing the disease burden. RTS,S/AS01 is a malaria vaccine candidate targeting the circumsporozoite protein and has proven efficacy in clinical trials.^5–10^ In July 2015, RTS,S/AS01 received a positive scientific opinion from the European Medicines Agency (EMA) under Article 58.^11^ In January 2016, the World Health Organization (WHO) recommended pilot implementation of RTS,S/AS01 in children 5–17 months of age.^12^ The large phase III clinical trial conducted in SSA in children aged 5–17 months and infants aged 6–12 weeks at first vaccination demonstrated vaccine efficacy (VE) against clinical malaria of 36.3% (95% confidence interval [CI] 31.8–40.5) in children and 25.9% (19.9–31.5) in infants, over a median follow-up of 48 and 38 months from the first vaccine dose in children and infants, respectively, in the modified intention-to-treat population. VE against severe malaria over the same follow-up period was 32.2% (13.7–46.9) in children and 17.3% (−9.4–37.5) in infants.^7^ Efficacy, immunogenicity, reactogenicity, and safety results from this trial have been reported previously.^7–10^ Here, we present more detailed safety results, both as defined per protocol and from *post-hoc* analyses. We further investigated the increased risk of febrile convulsions and the safety signals related to meningitis and cerebral malaria that were previously identified in this trial.^7–10,12,13^ In addition, analyses on gender-specific mortality and in specific subpopulations such as preterm infants and infants and children with low weight-for-age are described in detail.

## Results

### Study participants and reactogenicity

In total, 8922 children (enrolled at 5–17 months) and 6537 infants (enrolled at 6–12 weeks) were randomized to 1 of 3 regimens: 4 doses of RTS,S/AS01 (R3R group), 3 doses of RTS,S/AS01 followed by 1 dose of control vaccine (R3C group), or 4 doses of control vaccine (C3C group), administered at study months (M) 0, 1, 2, and 20. 8447 (95%) children and 6234 (95%) infants received the first 3 doses and 7384 (83%) children and 5488 (84%) infants received a fourth vaccine dose 18 months post-dose 3. 6187 (69%) children and 4637 (71%) infants completed the entire study.^7^ Children were followed up for a median of 48 months (interquartile range 39–50 months) and infants for 38 months (34–41 months) after the first vaccine dose. Baseline characteristics were similar in the 3 study groups.^7^ The mean age at dose 1 was 10.6–10.7 months across groups in the children cohort and 7.1–7.2 weeks in the infant cohort. Reactogenicity after the first 3 doses^8,9^ and post-dose 4^7^ has been previously reported (**Supplementary tables S1** and **S2**). On further investigation of the timing of the febrile reactions post-vaccination, we found that the increased incidence of fever (axillary temperature ≥37.5°C) following RTS,S/AS01 vaccination mainly occurred on the day after vaccination and fever mostly resolved within 1 day (**Supplementary figure S1**).

### Serious adverse events

The serious adverse event (SAE) incidences over the entire study period in the R3R, R3C, and C3C groups were 24.2%, 25.3%, and 28.4%, respectively, in children and 26.6%, 27.6%, and 28.4% in infants (Table 1). Across all groups and in children and infants, respectively, the most frequently reported SAEs were malaria (9.9%–14.2%; 8.3%–10.7%), pneumonia (6.8%–7.5%; 9.3%–10.0%), febrile convulsions (5.3%–6.2%; 4.1%–4.6%), gastroenteritis (5.0%–6.0%; 7.4%–7.9%), and anemia (4.2%–6.6%; 4.1%–5.3%).^7^ The incidence of SAEs that were judged by investigators to be causally related to vaccination ranged between 0.0% and 0.3% in both age categories, and most of them were fever-related.

**Table 1.** Serious adverse events.

	R3R		R3C		C3C	
	*N*	*% (95% CI)*	*N*	*% (95% CI)*	*N*	*% (95% CI)*
***At least one SAE (M0–SE)***						
5–17M (children)	2976	**24.2%** (22.7–25.8)	2972	**25.3%** (23.7–26.9)	2974	**28.4%** (26.8–30.1)
6–12W (infants)	2180	**26.6%** (24.8–28.5)	2178	**27.6%** (25.8–29.6)	2179	**28.4%** (26.5–30.4)
***At least one SAE (M0–SE), excluding malaria***						
5–17M (children)	2976	**22.6%** (21.1–24.2)	2972	**23.7%** (22.2–25.3)	2974	**26.4%** (24.8–28.0)
6–12W (infants)	2180	**25.8%** (24.0–27.7)	2178	**26.7%** (24.9–28.6)	2179	**27.1%** (25.3–29.0)
***At least one SAE within 30 days post–vaccination***						
5–17M (children)	2976	**6.1%** (5.3–7.0)	2972	**6.0%** (5.1–6.9)	2974	**6.7%** (5.8–7.6)
6–12W (infants)	2180	**4.8%** (4.0–5.8)	2178	**5.6%** (4.7–6.7)	2179	**5.2%** (4.3–6.2)
***At least one SAE before dose 4*** ***(M0–M20)***	**RTS,S/AS01**		**Control**			
5–17M, all children	5949	**18.6%** (17.6–19.6)	2974	**22.7%** (21.2–24.3)		
5–17M, low weight-for-age	695	**25.0%** (21.9–28.4)	364	**24.5%** (20.1–29.2)		
5–17M, very low weight-for-age	207	**26.6%** (20.7–33.1)	97	**28.9%** (20.1–39.0)		
6–12W, all infants	4358	**22.0%** (20.8–23.3)	2179	**23.1%** (21.3–24.9)		
6–12W, low weight-for-age	221	**28.5%** (22.7–34.9)	126	**30.2%** (22.3–39.0)		
6–12W, very low weight-for-age	147	**32.7%** (25.2–40.9)	67	**25.4%** (15.5–37.5)		
6–12W, preterm	244	**19.7%** (14.9–25.2)	118	**11.0%** (6.0–18.1)		
***At least one SAE post-dose 4*** ***(M21–SE)***	**R3R**		**R3C**		**C3C**	
5–17M, all children	2681	**10.3%** (9.2–11.5)	2719	**11.6%** (10.4–12.9)	2702	**10.6%** (9.5–11.8)
5–17M, low weight-for-age	277	**11.6%** (8.0–15.9)	304	**13.2%** (9.6–17.5)	297	**12.8%** (9.2–17.1)
5–17M, very low weight-for-age	48	**10.4%** (3.5–22.7)	50	**16.0%** (7.2–29.1)	60	**18.3%** (9.5–30.4)
6–12W, all infants	1966	**9.2%** (7.9–10.5)	1996	**9.7%** (8.4–11.1)	1976	**10.2%** (8.9–11.6)
6–12W, low weight-for-age	232	**14.7%** (10.4–19.9)	211	**10.0%** (6.3–14.8)	195	**12.3%** (8.0–17.8)
6–12W, very low weight-for-age	48	**12.5%** (4.7–25.2)	47	**19.1%** (9.1–33.3)	68	**22.1%** (12.9–33.8)
***At least one fatal SAE (M0–SE)***	**R3R**		**R3C**		**C3C**	
5–17M, all children	2976	**2.0%** (1.6–2.6)	2972	**1.7%** (1.3–2.3)	2974	**1.5%** (1.1–2.1)
5–17M, girls	1467	**2.4%** (1.7–3.3)	1500	**2.1%** (1.5–3.0)	1503	**1.1%** (0.7–1.8)
5–17M, boys	1509	**1.7%** (1.1–2.5)	1472	**1.3%** (0.8–2.0)	1471	**2.0%** (1.3–2.8)
6–12W, all infants	2180	**2.3%** (1.7–3.1)	2178	**2.5%** (1.9–3.3)	2179	**1.9%** (1.4–2.6)
6–12W, girls	1064	**2.5%** (1.7–3.7)	1060	**2.7%** (1.8–3.9)	1100	**1.5%** (0.8–2.4)
6–12W, boys	1116	**2.2%** (1.4–3.2)	1118	**2.3%** (1.5–3.4)	1079	**2.4%** (1.6–3.5)
***At least one fatal SAE within 30 days post-vaccination***	**R3R**		**R3C**		**C3C**	
5–17M (children)	2976	**0.3%** (0.1–0.6)	2972	**0.2%** (0.1–0.4)	2974	**0.2%** (0.1–0.4)
6–12W (infants)	2180	**0.6%** (0.3–1.0)	2178	**0.5%** (0.3–0.9)	2179	**0.3%** (0.1–0.6)

R3R, group receiving 4 doses of RTS,S/AS01; R3C, group receiving 3 doses of RTS,S/AS01 plus 1 dose of control vaccine; C3C, group receiving 4 doses of control vaccine; N, total number of children/infants in the group; %, percentage of children/infants with at least one SAE; M, months; W, weeks; SE, study end; CI, confidence interval; SAE, serious adverse event. Low weight-for-age defined as z-score ≤-2 but >-3, very low weight-for-age as z-score ≤-3.

The study population also included HIV-infected children and infants, although no systematic screening for HIV infection was performed. The proportion of participants identified and confirmed as HIV-infected between screening and study end (SE) was balanced across treatment groups: 0.9% in the control group and 1.0% in the RTS,S/AS01 groups for the combined age categories. Among HIV-infected children and infants, the safety profile appeared to be similar between RTS,S/AS01 and the control vaccine recipients. Detailed results for this subpopulation will be reported elsewhere (manuscript in preparation).

#### Fatal SAEs

No imbalance in fatal SAE incidence was observed between the treatment groups over the 30-day post-vaccination period and over the entire study period (CIs on group estimates overlapped) (Table 1). A total of 326 fatal SAEs were reported for children (R3R: 127 in 61 children; R3C: 94 in 51 children; C3C: 105 in 46 children) and 269 for infants (R3R: 85 in 51 infants; R3C: 104 in 55 infants; C3C: 80 in 42 infants). No fatality was considered by the investigators as related to vaccination. The most frequently reported fatal SAEs over the entire study period were malaria (0.3%–0.4%), pneumonia (0.2%–0.5%), gastroenteritis (0.2%–0.5%), anemia (0.2%–0.4%), and convulsions (0.3%) in the 5–17 months age group, and pneumonia (0.4%–0.7%), gastroenteritis (0.5%–0.6%), anemia (0.1%–0.6%), malaria (0.2%–0.4%), and sepsis (0.2%–0.3%) in the 6–12 weeks age group.

#### Safety in children and infants with low weight-for-age

The incidence of SAEs reported prior to dose 4 (M0–M20) and following the fourth dose (M21–SE) in children and infants with a low or very low weight-for-age at baseline (low: z-score ≤-2 but >-3; very low: z-score ≤-3) were in the same range in the RTS,S/AS01 and control groups (Table 1).

#### Safety in preterm infants

Safety up to M20 was evaluated in 362 infants who were born prematurely. The majority (approximately 90% in both groups) had a gestational age of 33–36 weeks; the 7 infants born with a gestational age below 30 weeks were all in the RTS,S/AS01 group (**Supplementary table S3**). SAE incidences in preterm infants were 19.7% in the RTS,S/AS01 and 11.0% in the control group (overlapping CIs) (Table 1).

Fatal SAEs were reported in 8 (3.3%; 95% CI: 1.4–6.4) preterm infants receiving RTS,S/AS01 (Fallot’s tetralogy, HIV infection, bronchopneumonia, pneumonia, and pneumococcal meningitis and sepsis) and 1 (0.8%; 0.0–4.6) preterm infant receiving control vaccine (HIV infection and tuberculosis). One fatal SAE occurred in an infant born at a gestational age <30 weeks. None of the fatal SAEs were considered related to vaccination.

### Adverse events of special interest

#### Febrile convulsions

During the 7-day post-vaccination period, a trend for a higher incidence of generalized convulsive seizures (level 1–3 of Brighton Collaboration Working Group [BCWG] diagnostic certainty) in RTS,S/AS01 vaccinees compared to controls was observed after the first 3 doses in children, and after the fourth dose in both children and infants (Table 2).^7,10^ The incidence of febrile convulsions within 7 days post-vaccination rose with increasing dose numbers, up to 2.5 per 1,000 doses in children and 2.2 per 1,000 doses in infants following the fourth dose in the R3R group. The distribution of time-to-onset of these cases indicated that febrile convulsions mainly happened within the first 2–3 days after dose 3 and 4 in children (**Supplementary Figure S2**).

This trend was confirmed in a *post-hoc* self-controlled case-series analysis of febrile convulsions in children who received any RTS,S/AS01 dose. This analysis showed an increased risk during the two assessed risk periods (within 3 and 7 days post-vaccination) compared with the respective control periods. The highest risk ratio was observed for the 3-day period (Table 2). This is consistent with the time window of febrile reactions following RTS,S/AS01.

**Table 2.** Febrile convulsions incidence and self-controlled case-series analysis.

Incidence per 1,000 vaccine doses of febrile convulsions with diagnostic certainty level 1–3 (Brighton collaboration), within 7 days after each dose						
	5–17months age category (children)			6–12 weeks age category (infants)		
	N	n	Incidence (95% CI)	N	n	Incidence (95% CI)
***First 3 doses***						
RTS,S/AS01	17,306	18	**1.0** (0.6–1.6)	12,739	2	**0.2** (0.0–0.6)
Control	8728	5	**0.6** (0.2–1.3)	6403	3	**0.5** (0.1–1.4)
***Dose 4***						
R3R	2447	6	**2.5** (0.9–5.3)	1825	4	**2.2** (0.6–5.6)
R3C	2472	3	**1.2** (0.3–3.5)	1837	0	**0.0** (0.0–2.0)
C3C	2473	1	**0.4** (0.0–2.3)	1827	1	**0.5** (0.0–3.0)
**SCCS Risk Ratio (95% CI) of febrile convulsions after each of the first 3 doses (5–17 months age category)**						
**Risk period**	**R3R**	**R3C**		**R3R+R3C**	**C3C**	
3 days (days 0–2)	**3.8** (1.7–8.7)	**2.3** (0.8–6.0)		**3.0** (1.6–5.6)	**0.8** (0.2–3.5)	
7 days (days 0–6)	**1.9** (0.9–4.2)	**2.2** (1.0–4.9)		**2.1** (1.2–3.6)	**1.1** (0.4–2.8)	
**SCCS Risk Ratio (95% CI) of febrile convulsions post-dose 4 (5–17 months age category)**						
**Risk period**	**R3R**	**R3C**			**C3C**	
3 days (days 0–2)	**6.0** (2.1–16.9)	**4.5** (0.4–49.6)			**0.0**	
7 days (days 0–6)	**2.9** (1.0–7.9)	**6.6** (0.6–72.5)			**0.4** (0.1–2.9)	

R3R, group receiving 4 doses of RTS,S/AS01; R3C, group receiving 3 doses of RTS,S/AS01 plus 1 dose of control vaccine; C3C, group receiving 4 doses of control vaccine; N, total number of vaccine doses administered in the group; n, number of vaccine doses followed by febrile convulsions; CI, confidence interval. Risk ratio is the risk ratio of the self-controlled case-series (SCCS) analysis, comparing the incidence in a given risk period (3 and 7 days post-vaccination) to the incidence in the corresponding control period (day 4–30 and day 8–30 post-vaccination, respectively).

However, the overall rate of febrile convulsions reported as SAE in children was not increased in the RTS,S/AS01 compared to the control group within 30 days post-vaccination (1.0% vs 0.8% following the first 3 doses; R3R: 1.1%, R3C: 0.9%, C3C: 1.1% following dose 4) or from M0–SE (R3R: 5.3%, R3C: 6.2%, C3C: 5.5%).

#### Rashes and mucocutaneous lesions

The incidence of rashes and mucocutaneous lesions occurring within 30 days post-vaccination in infants was balanced across groups (29.8% for the pooled RTS,S/AS01 group and 29.4% for the control group after the first 3 doses).

#### Potential immune-mediated disorders

During the entire study, 16 SAEs with Medical Dictionary for Regulatory Activities (MedDRA; version 18) terms included in the predefined list provided in the protocol (**Supplementary table S4**) were reported as potential immune-mediated disorder (pIMDs) (R3R: 8, R3C: 2, C3C: 6). None were considered causally related to vaccination. Review of the cases did not show any safety signals because no trends in time-to-onset after vaccination or type of pIMD were observed.

### Meningitis

**Figure 1.**
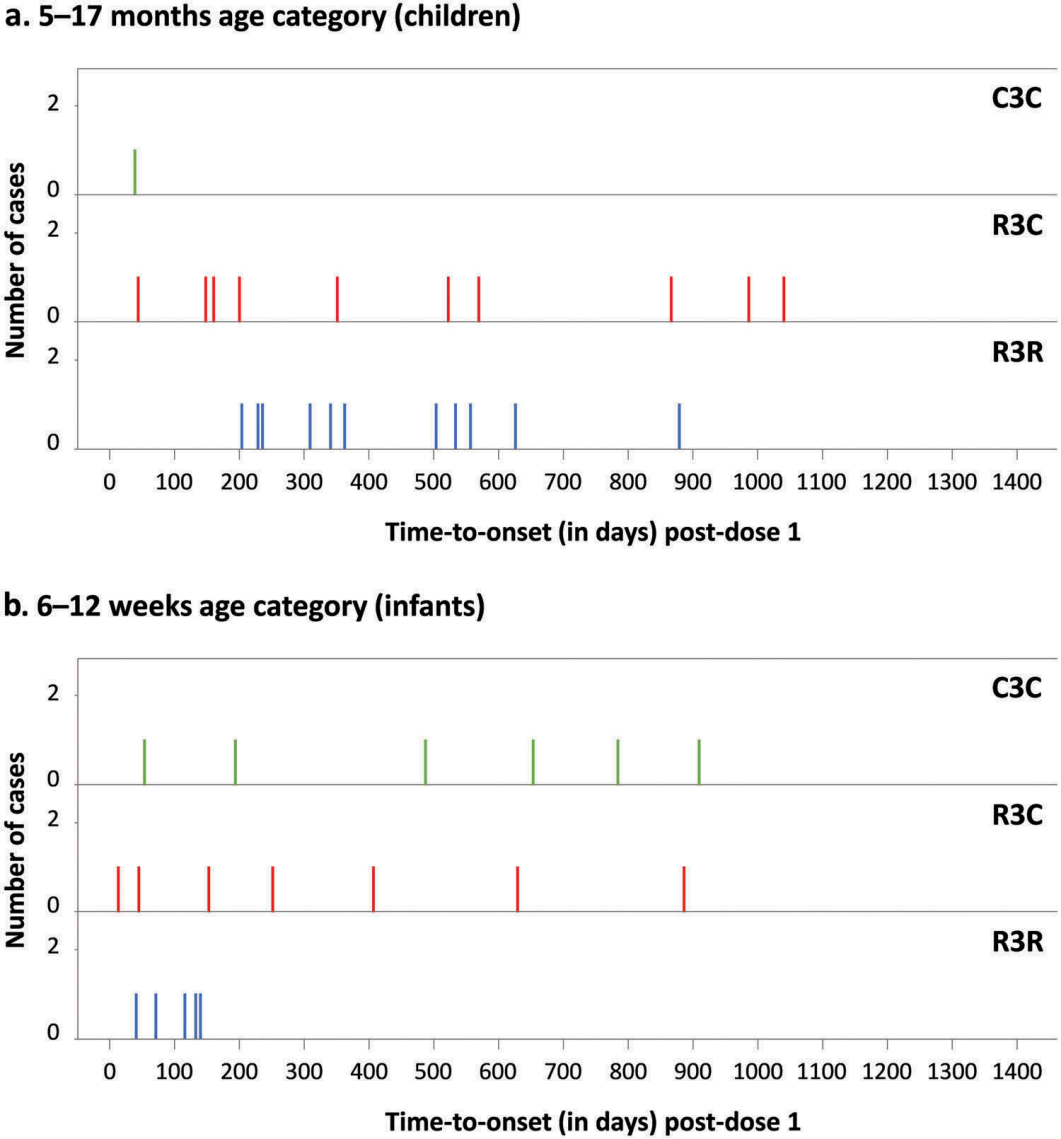
Meningitis cases by time-to-onset after dose 1 and by treatment group. R3R, group receiving 4 doses of RTS,S/AS01; R3C, group receiving 3 doses of RTS,S/AS01 plus 1 dose of control vaccine; C3C, group receiving 4 doses of control vaccine.

**Figure 2.**
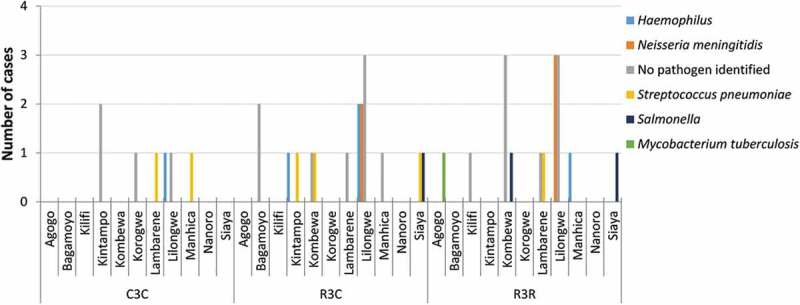
Distribution of meningitis cases by site and etiology in both age categories. R3R, group receiving 4 doses of RTS,S/AS01; R3C, group receiving 3 doses of RTS,S/AS01 plus 1 dose of control vaccine; C3C, group receiving 4 doses of control vaccine. One case with viral etiology is included in the “No pathogen identified” category (R3R group).

Following the primary analysis of the current study, an imbalance in the number of meningitis cases with any etiology was observed over the first year of follow-up post-dose 3.^9,10^ The analysis at SE showed that this imbalance in meningitis cases, which was considered a safety signal,^14^ persisted in the 5–17 months age group only.^7^ From M0–SE, 40 meningitis cases of any etiology were reported: 22 cases in children (R3R: 11, R3C: 10, C3C: 1) and 18 cases in infants (R3R: 5, R3C: 7, C3C: 6). The relative risk in children over the entire study period was statistically significant: 11.0 for R3R vs C3C and 10.0 for R3C vs C3C (Table 3). When assessed over follow-up periods from dose 1 up to 12 months or 18 months post-dose 3, the relative risk (R3R+R3C vs C3C) in children was 5.5 and 8.0, respectively.^8,10^ Following the fourth RTS,S/AS01 dose only 1 case in children and no cases in infants were reported. Across all treatment groups and both age categories, 58% of cases (23/40) occurred within 12 months after the first 3 doses; 55% of the cases in children (12/22; R3R: 6, R3C: 5, C3C: 1) and 61% of the cases in infants (11/18; R3R: 5, R3C: 4, C3C: 2). However, no cluster in time-to-onset was observed and cases occurred sporadically throughout the study (Figure 1) and over the entire age range of the participants (**Supplementary figure S3**). Of all meningitis cases, 38% (15/40) were reported in Lilongwe, Malawi, but no clustering in time or etiology indicated an outbreak in this region (Figure 2 **and Supplementary figure S4**). Pathogens were identified in 53% (21/40) of meningitis cases: 20 cases with bacterial pathogens and 1 viral meningitis case (Table 3).

**Table 3.** Meningitis cases.

Meningitis cases based on clinical diagnosis by treating physician, n						
	**5–17 months age category (children)**			**6–12 weeks age category (infants)**		
**Before dose 4 (M0–M20)**						
	**R3R**	**R3C**	**C3C**	**R3R**	**R3C**	**C3C**
	**N = 2976**	**N = 2972**	**N = 2974**	**N = 2180**	**N = 2178**	**N = 2179**
Meningitis (no pathogen identified)	4	5	1	2	2	2
Meningitis haemophilus	1	0	0	0	0	0
Meningitis meningococcal	3	1	0	0	0	0
Meningitis pneumococcal	0	1	0	1	2	1
Meningitis tuberculous	0	0	0	0	0	0
Meningitis viral	1	0	0	0	0	0
Meningitis salmonella	0	0	0	2	1	0
***Meningitis total***	***9***	***7***	***1***	***5***	***5***	***3***
**Post-dose 4 (M21–SE)**						
	**R3R**	**R3C**	**C3C**	**R3R**	**R3C**	**C3C**
	**N = 2681**	**N = 2719**	**N = 2702**	**N = 1996**	**N = 1996**	**N = 1976**
Meningitis (no pathogen identified)	1^a^	0	0	0	1	1
Meningitis haemophilus	0	2	0	0	1	1
Meningitis meningococcal	0	1	0	0	0	0
Meningitis pneumococcal	0	0	0	0	0	1
Meningitis tuberculous	1	0	0	0	0	0
Meningitis viral	0	0	0	0	0	0
Meningitis salmonella	0	0	0	0	0	0
***Meningitis total***	***2***	***3***	***0***	**0**	**2**	**3**
**Relative risk for meningitis**						
	**5–17 months age category (children)**			**6–12 weeks age category (infants)**		
*Before dose 4 (M0–M20)*	R3R+R3C vs C3C:			R3R+R3C vs C3C:		
	8.0 (95% CI: 1.1–60.3)*^b^*			1.5 (95% CI: 0.4–5.6)*^b^*		
*Entire study (M0–SE)*	R3R vs C3C: 11.0 (95% CI: 1.4–85.1)			R3R vs C3C: 0.8 (95% CI: 0.3–2.7)		
	R3C vs C3C: 10.0 (95% CI: 1.3–78.1)			R3C vs C3C: 1.2 (95% CI: 0.4–3.5)		
**Meningitis and other central nervous system infections assessed by external experts, n**						
	**5–17 months age category (children)**			**6–12 weeks age category (infants)**		
**Before dose 4 (M0–M20)**						
	**R3R**	**R3C**	**C3C**	**R3R**	**R3C**	**C3C**
Confirmed meningitis	5	4	0	3	4	1
No meningitis	3	2	2	3	2	3
Undetermined	4	2	3	2	0	0
**Post-dose 4 (M21–SE)**						
	**R3R**	**R3C**	**C3C**	**R3R**	**R3C**	**C3C**
Confirmed meningitis	1	2	0	0	1	2
No meningitis	1	2	0	0	0	1
Undetermined	1	0	0	0	1	0

R3R, group receiving 4 doses of RTS,S/AS01; R3C, group receiving 3 doses of RTS,S/AS01 plus 1 dose of control vaccine; C3C, group receiving 4 doses of control vaccine; N, total number of children/infants per group; n, number of cases within the specified category; CI, confidence interval; M, month; SE, study end.

^a^ This case occurred more than 20 months after dose 1, however, dose 4 was not given.

^b^ RTS,S Clinical Trials Partnership PLoS Med 2014; 11: e1001685.

Confirmed meningitis, if the clinical pictures and the lab results were consistent with diagnosis of meningitis; undetermined, if the clinical pictures and/or the lab results were not consistent with diagnosis of meningitis and not consistent with another diagnosis, or were missing; no meningitis, if the clinical pictures and the lab results were consistent with another diagnosis.

To further analyze the safety signal for meningitis, a larger number of SAEs (n = 55) including all central nervous system (CNS) infection/inflammation terms were reviewed by two independent external experts. Among these 55 CNS infection/inflammation cases (including the 40 meningitis cases reported by the investigators), 23 were confirmed as meningitis (Table 3), all of which were previously diagnosed as meningitis by the investigators. Of the 17 meningitis cases reported by the investigators that were not confirmed by the experts, 10 had occurred in children and 7 in infants. The imbalance between groups in the 5–17 months age category remained among meningitis cases confirmed by external experts.

### Cerebral malaria

**Figure 3.**
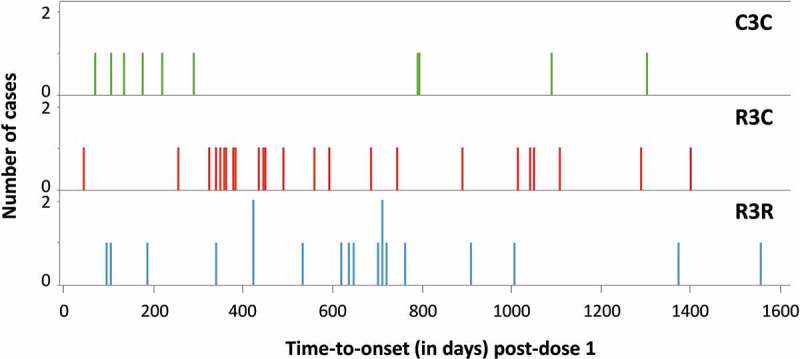
Cerebral malaria cases (identified per computer algorithm) by time-to-onset after dose 1 and by treatment group (5–17 months age category). R3R, group receiving 4 doses of RTS,S/AS01; R3C, group receiving 3 doses of RTS,S/AS01 plus 1 dose of control vaccine; C3C, group receiving 4 doses of control vaccine.

To further investigate the previously observed increased risk of severe malaria between M21 and SE in children in the R3C group compared to the C3C and R3R groups,^7^ we retrospectively looked in more detail into hospitalized severe malaria cases. This analysis revealed a safety signal for cerebral malaria (CM).^12,13^ For this analysis, we used a computer algorithm to identify cases with parasitemia >5,000/µL and a Blantyre coma score (BCS) ≤2 as a proxy for cerebral malaria (CM), regardless of whether the CM clinical diagnosis was confirmed by the investigators and without excluding children with comorbidities. Among the 53 cases identified as CM by this algorithm in the 5–17 months age group (M0–SE), statistically significantly more CM cases were identified in children vaccinated with RTS,S/AS01 compared to control (R3R: 19, R3C: 24, C3C: 10; p value: 0.0276 using Fisher exact test) (Table 4). This was not observed in infants (R3R: 6, R3C: 7, C3C: 7). No well-defined clustering in time-to-onset after vaccination was observed (Figure 3). Of note, an imbalance in CM cases was also observed before dose 4 (M0–M20) when comparing the R3R group (7 cases) and R3C group (15 cases) even though these children received the same vaccines up to that point (Table 4).

**Table 4.** Distribution of severe malaria manifestations.

Hospitalized severe malaria* classified by specific markers of severe disease, n (%)						
	5–17 months age category (children)			6–12 weeks age category (infants)		
**Before dose 4 (M0–M20)**						
	**R3R**	**R3C**	**C3C**	**R3R**	**R3C**	**C3C**
	**N = 86**	**N = 119**	**N = 158**	**N = 82**	**N = 66**	**N = 86**
**BCS ≤2 (compatible with CM)**	**7 (8.1)**	**15 (12.6)**	**6 (3.8)**	**2 (2.4)**	**3 (4.5)**	**4 (4.7)**
Hb ≥5 g/dL	6 (7.0)	10 (8.4)	5 (3.2)	0 (0.0)	2 (3.0)	3 (3.5)
Hb <5 g/dL	1 (1.2)	5 (4.2)	1 (0.6)	2 (2.4)	1 (1.5)	1 (1.2)
**Hb <5 g/dL (and BCS >2)**	11 (12.8)	14 (11.8)	29 (18.4)	13 (15.9)	17 (25.8)	17 (19.8)
**Other**	67 (77.9)	90 (75.6)	123 (77.9)	65 (79.3)	46 (69.7)	65 (75.6)
**Missing**	1 (1.2)	0 (0.0)	0 (0.0)	2 (2.4)	0 (0.0)	0 (0.0)
**Post-dose 4 (M21–SE)**						
	**R3R**	**R3C**	**C3C**	**R3R**	**R3C**	**C3C**
	**N = 76**	**N = 103**	**N = 76**	**N = 53**	**N = 63**	**N = 68**
**BCS ≤2 (compatible with CM)**	**12 (15.8)**	**9 (8.7)**	**4 (5.3)**	**4 (7.6)**	**4 (6.4)**	**3 (4.5)**
Hb ≥5 g/dL	11 (14.5)	9 (8.7)	2 (2.6)	4 (7.6)	4 (6.4)	2 (3.0)
Hb <5 g/dL	1 (1.3)	0 (0.0)	2 (2.6)	0 (0.0)	0 (0.0)	1 (1.5)
**Hb <5 g/dL (and BCS >2)**	11 (14.5)	18 (17.5)	17 (22.4)	15 (28.3)	15 (23.8)	19 (27.9)
**Other**	53 (69.7)	75 (72.8)	54 (71.1)	34 (64.2)	42 (66.7)	45 (66.2)
**Missing**	0 (0.0)	1 (1.0)	1 (1.3)	0 (0.0)	2 (3.2)	1 (1.5)
**Cerebral malaria following external expert adjudication (5–17 months age category), n**						
	**Before dose 4 (M0–M20)**			**Post-dose 4 (M21–SE)**		
	**R3R**	**R3C**	**C3C**	**R3R**	**R3C**	**C3C**
	**N’ = 52**	**N’ = 69**	**N’ = 75**	**N’ = 40**	**N’ = 60**	**N’ = 44**
**Possible CM**	3	10	7	7	8	2
Confirmed (by both experts)	2	4	3	2	6	1
Confirmed (by 1 expert)	0	2	1	1	0	1
Uncertain (by 1 or both experts)	1	4	3	4	2	0
**No CM**	49	59	68	33	52	42

R3R, group receiving 4 doses of RTS,S/AS01; R3C, group receiving 3 doses of RTS,S/AS01 plus 1 dose of control vaccine; C3C, group receiving 4 doses of control vaccine; N, number of children/infants hospitalized with severe malaria, *secondary case definition 1 (>5,000 parasites/µL and at least one marker of severe disease, without exclusion of comorbidities); N’, number of children hospitalized with severe malaria with >0 parasites/µL and at least one neurological marker of severe disease, i.e., BCS ≤2, prostration or 2 or more seizures; n (%), number (percentage) of cases within the specified category; M, month; SE, study end; CM, cerebral malaria; BCS, Blantyre coma score; Hb, hemoglobin; other, BCS >2 and Hb ≥5.0 g/dL and at least one of the following: prostration, respiratory distress, 2 or more seizures, hypoglycemia <2.2 mmol/L, acidosis base excess ≤-10.0 mmol/L, or lactate ≥5.0 mmol/L; missing, Hb or BCS unavailable so syndrome classification could not be determined; possible CM, at least one expert could not exclude a diagnosis of CM, i.e., classified the case as “Confirmed CM” or as “Uncertain CM”; no CM, both experts classified the case as “Not CM”; uncertain, both experts classified the case as “Uncertain CM” or one expert classified the case as “Uncertain and one as “Not CM”.

To gain understanding of the CM signal, 340 hospitalized severe malaria cases with parasitemia >0/µL and with at least one neurological marker of severe disease (BCS ≤2, or seizure, or prostration) were reviewed retrospectively by two independent external experts. Among these cases, there were 37 for which at least one expert could not rule out a diagnosis of CM and which were considered as possible CM cases. Of these 37 cases, 23 were classified as “confirmed CM” by at least one expert. For 18 of these, both experts agreed it was CM (Table 4). 45% (24/53) of the CM cases identified by the computer algorithm were considered as “not CM” by the experts, and 8 new cases considered as possible CM by the experts had not been identified as CM by the algorithm. The same pattern, in terms of study period (M0–M20 or M21–SE) and treatment group distribution, was observed for the cases identified by the experts as for the ones previously identified by the computer algorithm.

### Gender-specific mortality

As previously reported,^15^ all-cause mortality in girls (all ages, M0–SE) who received RTS,S/AS01 (123/5091 [2.4%; 2.0–2.9]) was higher than in girls who received control vaccine (33/2603 [1.3%; 0.9–1.8]), a difference not observed in boys (95/5215 [1.8%; 1.5–2.2] vs 55/2550 [2.2%; 1.6–2.8]). Since long-term outcomes such as death are multifactorial and prone to confounding, mortality in the trial population was evaluated *post-hoc* by modeling known risks factors by Cox regression. Explanatory variables included in the model were RTS,S/AS01 vaccination, gender, age category, trial site, HIV status (which was not systematically collected in all participants), baseline anemia, distance from inpatient facility, and low weight-for-age at baseline. As the effect of RTS,S/AS01 vaccination on mortality was observed to be modified by gender (p value: 0.0014), we performed subgroup analyses by gender. In girls, mortality was different across trial sites and increased mortality was associated with HIV positivity, low weight-for-age, baseline anemia, RTS,S/AS01 vaccination (3 or 4 doses vs control), and the younger age cohort (6–12 weeks vs 5–17 months) (**Supplementary table S5**). Except for RTS,S/AS01 vaccination and baseline anemia, the same risk factors were observed for boys (**Supplementary table S5**). No clustering in time to death after vaccination was observed (not shown). We found no obvious pattern in the cause of death that could explain the gender-specific imbalance in mortality (**Supplementary table S6**).

### Discussion

We have previously reported that a 4-dose regimen of RTS,S/AS01 was efficacious and provided the strongest benefit in terms of impact against clinical and severe malaria in children aged 5–17 months at the time of the first vaccination.^16^ Additionally, reductions in overall hospital admissions, admissions because of malaria, severe anemia, and the need for blood transfusion in children were observed. We also previously reported an increased risk of febrile convulsions in children vaccinated with RTS,S/AS01 in our large double-blind, randomized, phase III trial in SSA.^7,10^ In addition, two safety signals were identified in this trial: an increased number of meningitis and cerebral malaria cases in RTS,S/AS01-vaccinated children compared to controls.^7–10,12,13^ In this manuscript, we reported more detailed results on these safety signals and described safety outcomes in specific subpopulations in the trial.

There was no imbalance between the groups in terms of percentages of children and infants with adverse events (AEs) and SAEs, except those that are discussed below. Of note, no difference was observed for sepsis or pneumonia cases, which were assessed as secondary objectives of this study.^7^

Febrile convulsions form a particular subgroup of generalized convulsive seizures that occur in 5% of children and can occur with different infectious diseases including malaria.^17^ Febrile convulsions are the most common seizures occurring after vaccination. An increased incidence of febrile convulsions was observed during the first 2–3 days after RTS,S/AS01 vaccination in children 5 months or older. The higher risk for febrile convulsions following dose 3 and 4 is in line with the expected age distribution of febrile convulsions: children aged 3 months to 6 years,^18^ with a peak at 18 months.^19^ The increased risk of febrile convulsions during the first 2–3 days after RTS,S/AS01 vaccination is consistent with the risk period for post-vaccination febrile reactions observed in this study (mostly on the day after vaccination) and confirms that convulsions occurring soon after RTS,S/AS01 administration were mostly triggered by vaccination-induced fever, as observed with other pediatric vaccines.^18,20–25^ No imbalance was observed over the 30-day post-vaccination period or from M0–SE, possibly because of the reduction of malaria episodes and associated malaria-related febrile seizures in the RTS,S/AS01-vaccinated children.

An imbalance in the number of meningitis cases was previously noted in this trial, with meningitis being more frequently reported among children in the RTS,S/AS01 groups compared to control children before the fourth dose.^8,10^ While this imbalance persisted in children over the entire follow-up,^7^ it was not observed when comparing the R3R with the R3C group after the fourth vaccine dose. The etiology of meningitis cases was heterogeneous, including different pathogens common in this population. No cluster in time-to-onset after vaccination that might suggest a temporal relationship between vaccination and the occurrence of meningitis nor a temporal clustering of cases that might suggest an outbreak of epidemic meningitis was observed throughout the whole follow-up. Furthermore, 38% of cases were reported in one study site (Lilongwe, Malawi), but were not associated with any recognized meningitis outbreak in Malawi during the study period. Differences in the number of meningitis cases reported by the investigators (n = 40) compared to those confirmed by the external experts (n = 23) are related to the more stringent case definition requiring consensus of both experts, and the fact that investigators also reported suspected meningitis cases without laboratory confirmation. An imbalance of meningitis has not been observed in other RTS,S/AS01 trials.^26^ The available pre-clinical toxicology and bio-distribution data from animal studies on RTS,S and AS01 did not reveal any treatment-related clinical or histological signs of meningitis, encephalitis, convulsion, neurotoxicity, or inflammation of the brain in any of the examined animals.^27,28^ It is currently unknown if a causal link between the vaccine and meningitis is biologically plausible. The most likely hypothesis to explain the meningitis signal seems to be a chance finding, due to the single case of meningitis in the control group of approximately 3000 children followed for almost 4 years. While there are no robust estimates of background incidence rates of meningitis in SSA, it is striking to see that, in infants, 6 meningitis cases were observed among the control group of approximately 2000 infants followed for 3 years, of which 4 occurred at an age of 10 months or older (**Supplementary figure S3**). The hypothesis of a probable chance finding was also pointed out by investigators, the independent data monitoring committee, consulted external experts, WHO, and EMA.^12,29^ The meningitis signal will nevertheless be closely monitored during phase IV studies and the pilot implementations. Details on the phase IV studies can be found on gsk-studyregister.com (study identifiers: 115055, 115056, 116682) and ClinicalTrials.gov (NCT02374450, NCT02251704).

Cerebral malaria cases were not clinically confirmed by the investigators; they were identified by a highly sensitive, but possibly poorly specific computer algorithm based on the coma score. Severe malaria cases with any neurological marker of severe disease were therefore reviewed by two external experts, who identified fewer possible CM cases (n = 37, including some new cases) compared to the computer algorithm (n = 53). Nevertheless, the distribution across the groups was similar to that identified with the algorithm, with more CM cases noted in children (5–17 months age category) who received any dose of RTS,S/AS01 compared to the control group. The time-to-onset of the CM cases did not show a well-defined clustering that might indicate a direct effect of the vaccine or an indirect effect related to rebound. Currently we have identified no biologically plausible explanation for how a pre-erythrocytic vaccine could directly affect the pathogenesis of severe disease occurring after the blood-stage infection. Theoretically, an increase in CM could be a result of delayed exposure to malaria and delayed acquired immunity in the setting of ongoing malaria transmission. Any malaria intervention has the potential to result in such a rebound effect, especially in areas of high transmission. However, available evidence on a possible rebound effect due to RTS,S/AS01 vaccination is inconclusive.^30–33^

Another hypothetical explanation for the observed imbalance in meningitis and CM cases may be that the low number of cases in the control group in the 5–17 months age category may be due to a non-specific protective effect on CNS infections of the rabies control vaccine these children received.^34^ Further studies would be needed to investigate the hypothesis of non-specific vaccine effects.

The safety profile in preterm infants and children with low weight-for-age was similar to that in the global study population. There was a trend for a higher proportion of preterm infants with SAEs and fatal SAEs. However, none of these SAEs was deemed related to vaccination and the study was not designed to specifically assess safety in preterm infants. There was no indication of an increased risk in children with malnutrition and therefore, no indication that these children should be excluded from future immunization programs.

Despite the significant reductions in severe malaria, severe malarial anemia, and blood transfusions, which serve as proximal markers of mortality reduction, no significant effect of RTS,S/AS01 vaccination on all-cause mortality was noted in our trial.^7^ This may be due to the close follow-up, access to quality care, and prompt treatment of all participants enrolled in this phase III study when necessary, but could also be due to the limited power to detect an effect on overall mortality. Malaria is a treatable disease and mortality from severe malaria can largely be avoided with prompt antimalarial treatment and, when required, blood transfusion.^35–38^ In a case-control study in one of our study sites, children participating in this trial had a 70% lower mortality, regardless of the study group, compared to the general population,^39^ likely due to the high standard of care provided in context of the trial. The childhood deaths that occurred in this study were therefore unlikely to have been representative of those that occur in the general pediatric population. A lack of impact on mortality in phase III trials has also been observed for other vaccines; no reduction in the number of deaths was seen in children vaccinated with the oral, live attenuated human rotavirus vaccine compared to placebo-vaccinated children.^40^ However, a significant decline in diarrhea-related deaths was observed after introduction of rotavirus vaccination in Mexican children.^41^

In this context, the apparent increased all-cause mortality in RTS,S/AS01-vaccinated girls compared to control girls in our study^15^ needs to be interpreted with caution. Various causes of death were reported during the trial, including trauma, malaria, and other infectious diseases; and no deaths were considered as related to vaccination by the investigators or physicians caring for the children in the study. Mortality in the trial population was evaluated by modeling known risks factors, but information on some of these risk factors was not indicated per protocol, and therefore not systematically collected. Exploratory analyses revealed that mortality was independently associated with a number of known risk factors (HIV infection, malnutrition, anemia, young age). Although none of these factors explained the apparent excess mortality in female RTS,S/AS01 recipients, the multiple risk factors for death identified and not accounted for during randomization underscores the risk of such *post-hoc* analyses. The increased risk for mortality in RTS,S/AS01-vaccinated girls could be the result of the observed low female mortality rate in the control arm, which at 1.3% was lower than the mortality rate in all other study arms, both male and female. We do not have data from the trial on gender-specific parental care-seeking behaviors,^42^ which might have confounded these gender-specific mortality outcomes.

Potential limitations of the study include the fact that the self-controlled case-series for febrile convulsions, gender-specific mortality, and CM cases were analyzed *post-hoc* and those analyses were data-driven. These results therefore need to be interpreted with caution. Furthermore, there was no clinical diagnosis performed for CM as the investigators were not asked to specifically report CM. Another limitation might be the fact that, in some cases, children had been treated for malaria before admission to hospital, which could have influenced the final diagnosis, especially for CM. Also, the overlap of clinical symptoms for meningitis and CM and the low proportion of cases for which we were able to identify the causative pathogens of meningitis might have led to over-reporting of cases; this has been evident from the review of cases by the experts. In addition, the analyses in the different subpopulations were limited by the low number of participants in these subpopulations and the fact that the trial was not specifically designed to assess safety in these subgroups. Finally, while the sample size of our trial was large, it may not have been sufficient to detect rare or very rare AEs after RTS,S/AS01 vaccination.

In conclusion, in this trial, RTS,S/AS01 has demonstrated a positive benefit-risk balance confirming the previous findings. Febrile convulsions are an identified risk of RTS,S/AS01, similar to other pediatric vaccines.^43^ The higher number of meningitis and cerebral malaria cases in the RTS,S/AS01 groups are not considered confirmed risks, but rather potential risks given the low likelihood of these events being caused directly by the vaccine, the absence of a cluster in time-to-onset after vaccination, and the low number of cases. These safety signals may be chance findings but – given their severity – will be further investigated. The imbalance in mortality between RTS,S/AS01-vaccinated and control girls could be related to currently unknown non-specific vaccine effects but should be interpreted with caution due to the overall low mortality rate in the trial. At present, the impact of RTS,S/AS01 on mortality is classified as missing information in the risk management plan.^44^

Based on the positive benefit-risk balance of RTS,S/AS01 and the potential for substantial impact against clinical and severe malaria, EMA granted a positive scientific opinion for this vaccine, and WHO recommended pilot implementation of the vaccine in children in sub-Saharan Africa.^11,12^ The identified safety signals will be closely monitored during this pilot implementation and in phase IV studies. These studies will also provide additional real-life data which cannot be generated in a pre-licensure clinical trial setting.

**Figure 4.**
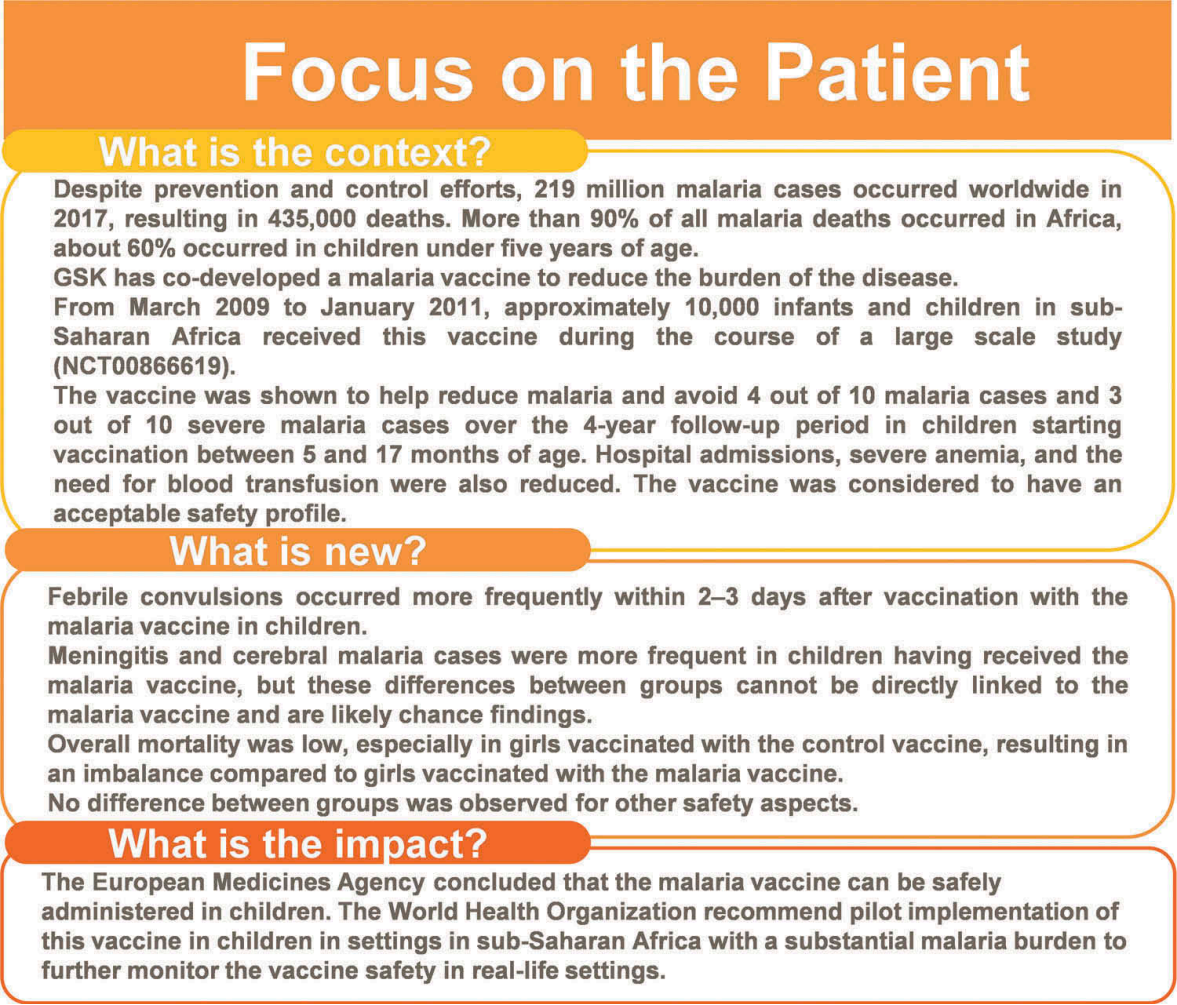
Focus on the patient summary of the findings.

Figure 4 represents a “focus on the patient” section, which elaborates on the clinical relevance of the research intended to be shared with patients by health care professionals.

## Patients and methods

### Study design and ethics

This phase III, double-blind, randomized, controlled trial (ClinicalTrials.gov: NCT00866619) was conducted between March 2009 and January 2014 at 11 centers in 7 SSA countries: Burkina Faso, Ghana, Gabon, Kenya, Tanzania, Malawi, and Mozambique. Detailed trial methods have been reported previously.^7–10,45–48^ The study was conducted in accordance with Good Clinical Practice guidelines (**Supplementary methods**) and cleared by all institutional review boards of the countries and centers concerned.

The trial was overseen by an Independent Data Monitoring Committee (**Supplementary methods**).

### Randomization and blinding

Children 5–17 months and infants 6–12 weeks of age were randomly assigned (1:1:1 ratio) to receive 4 doses (M0, 1, 2, 20) of RTS,S/AS01 (R3R group) or control vaccine (C3C; control group), or 3 RTS,S/AS01 doses (M0, 1, 2) plus control vaccine (M20) (R3C group). Data were collected in an observer-blind manner; caregivers of participants, and those responsible for evaluation of study endpoint data were unaware which treatment was administered.

### Study vaccines

RTS,S/AS01 (GSK, Rixensart, Belgium) contains RTS,S antigen combined with AS01_E_ Adjuvant System (**Supplementary methods**). As control vaccine, infants received meningococcal serogroup C conjugate vaccine (MenC-CRM, *Menjugate*, Novartis, Basel, Switzerland), while older children received 3 doses of rabies vaccine (*Verorab*, Sanofi Pasteur, Paris, France), followed by 1 dose of MenC-CRM. Infants received study vaccines concurrently with routine pediatric vaccines according to each country’s Expanded Program on Immunization.

### Safety assessment per protocol

SAEs were collected throughout the trial by passive surveillance from M0 to SE for all participants. Solicited symptoms were collected during a 7-day post-vaccination period and unsolicited AEs were collected during a 30-day post-vaccination period among the first 200 participants per age group at each study site. All hospitalized participants were evaluated for severe malaria based on predefined markers of severe disease and a protocol-defined algorithm (**Supplementary methods**). Verbal autopsies were conducted on deaths that occurred outside study facilities.^49^ The cause of death was determined by a panel of three experienced verbal autopsy reviewers, as detailed in the **Supplementary methods**.

The investigators used their clinical judgement to determine the relationship between the vaccine and the occurrence of each (S)AE (**Supplementary methods**).

Individual medical review was performed for all SAEs. Aggregate safety data were reviewed by a multi-functional team consisting of all the appropriate disciplines required to assess any potential safety issue by the trial sponsor. This team met on a regular basis and when information arose that could potentially impact the benefit-risk of the vaccine.

Safety (SAEs) was also evaluated in infants and children with low and very low weight-for-age and in preterm infants (<37 weeks of gestational age). While there were no protocol-defined objectives pertaining to the safety analysis of preterm infants, these data were readily retrievable since the protocol-defined procedures called for recording gestation in study participants in the 6–12 weeks age category. Gestation at birth was solicited by asking the parent(s)/LAR(s) if their infant was born in the 37^th^ week of gestation or later. For infants born before the 37^th^ week, the approximate duration of gestation (in weeks) at birth, as provided by the parent(s)/LAR(s), was recorded.

AEs of specific interest included convulsions, rashes and mucocutaneous lesions assessed within 30 days following vaccination, and pIMDs from M0–SE. The specific interest in the detailed description of convulsions, rashes and mucocutaneous lesions resulted from an imbalance in these AEs observed in a pooled analysis of safety data from phase II RTS,S trials.^26^ pIMDs were considered of specific interest based on the theoretical concern that administration of an adjuvanted vaccine may interfere with immunological self-tolerance.

All convulsions occurring within 30 days post-vaccination were reported as SAEs. Those occurring within 7 days post-vaccination were recorded and analyzed according to the BCWG case definition for general convulsive seizures.^18^ Those occurring after the 7-day post-vaccination period were diagnosed and reported by the clinicians as any other SAE.

Rashes and mucocutaneous lesions that occurred within 30 days of vaccination in the first 200 participants enrolled at each site in the infant age group were reported as AEs/SAEs and analyzed according to the BCWG case definition.^50^ Medical documentation of the events was reported in the electronic case report forms. Rashes and mucocutaneous lesions that met the criteria for an SAE were reported in all participants throughout the study period.

pIMDs were recorded as SAEs (M0–SE) according to a predefined list provided in the protocol (**Supplementary table S4**). Additionally, any other AE that could be immune-mediated in the investigator’s judgment had to be reported according to the same process. Study investigators were offered facilitated access to laboratory investigation. The analysis was performed based on the predefined list of MedDRA preferred terms and on the investigator’s judgment.

Safety analyses were performed on the intention-to-treat population, which included all participants who received at least 1 vaccine dose. Statistical evaluations of safety endpoints were descriptive. Proportions of children and infants for whom (S)AEs were reported were tabulated with exact 95% CIs.

### Post-hoc *analyses*

A self-controlled case series *post-hoc* analysis was applied on febrile convulsions within 30 days after any dose in children. The risk ratio was calculated as the ratio of incidence of events during a risk period, to the incidence during a control period. The two risk periods were 3 and 7 days post-vaccination (days 0–2 and 0–6 with day 0 being the day of vaccination), resulting in control periods of 27 days (days 3–29) and 23 days (days 7–29) post-vaccination.

To further assess the safety signal for meningitis identified in this study, available cerebrospinal fluid samples from suspected meningitis cases were sent to an external laboratory to perform PCR analysis (**Supplementary methods**). A larger number of SAEs including all CNS infection/inflammation terms were reviewed by two independent external experts, individually and in a blinded way. The experts independently categorized the cases as: “confirmed meningitis” if the clinical picture and the laboratory results were consistent with a diagnosis of meningitis; “no meningitis” if the clinical picture and laboratory results were consistent with another diagnosis; “undetermined” if the clinical picture or laboratory results were not consistent with a diagnosis of meningitis and not consistent with another diagnosis, or were missing. The final categorization of cases required consensus of both experts **(Supplementary methods)**.

In a *post-hoc* analysis, hospitalized severe malaria cases (secondary case definition 1 with parasitemia >5,000/µL;^10^ **Supplementary methods**) with BCS ≤2 were identified from inpatient records by a computer algorithm as a proxy for CM, regardless of the presence of comorbidities or whether the diagnosis was clinically confirmed by the investigators. To complement this analysis with clinical expertise, two independent experts in malaria reviewed all hospitalized severe malaria cases in the 5–17 months age category with parasitemia >0/µL and with a BCS of ≤2, or seizure, or prostration to retrospectively confirm and/or identify any CM case. The experts categorized the cases as “confirmed CM”, if the case fulfilled the CM case definition (adapted from the WHO)^51^: *“The clinical syndrome characterized by coma (unable to localize a painful stimulus, or BCS <3) maintained for a minimum of 30 minutes after a convulsion or the correction of hypoglycemia, in the presence of asexual P. falciparum parasites in peripheral blood (>0 parasites/µL), having excluded other causes of encephalopathy (including meningitis)”*; “uncertain CM” if the case was not sufficiently documented to either confirm or rule out CM diagnosis; or “not CM” if the case was more likely due to another etiology/diagnosis (**Supplementary methods**).

We performed a *post-hoc* multivariate Cox regression analysis to model risk factors known to be associated with mortality.

### Trademark statement

Menjugate is a trademark of Novartis.

Verorab is a trademark of Sanofi Pasteur.

## Figures and Tables

**Table:** 

AE	adverse event
AS	adjuvant system
BCS	Blantyre coma score
BCWG	Brighton Collaboration Working Group
CI	confidence interval
CM	cerebral malaria
CNS	central nervous system
EMA	European Medicines Agency
HIV	human immunodeficiency virus
M	month
MedDRA	Medical Dictionary for Regulatory Activities
PCR	polymerase chain reaction
pIMD	potential immune-mediated disorder
SAE	serious adverse event
SE	study end
SSA	sub-Saharan Africa
VE	vaccine efficacy
WHO	World Health Organization
