# Supplementary material for: Safety profile of the RTS,S/AS01 malaria vaccine in infants and children: additional data from a phase III randomized controlled trial in sub-Saharan Africa
Source: Hum Vaccin Immunother. 2019 Apr 23;15(10):2386–98. doi: 10.1080/21645515.2019.1586040 (PMC6816384; doi:10.1080/21645515.2019.1586040)
Supplement: Supplemental Material [file khvi-15-10-1586040-s001.zip › Mal-055 safety mns_25JAN2019_clean_Supplement_Figures.docx]

**Figure S1. Incidence of fever on each day after the 4 doses (ITT population)**

**
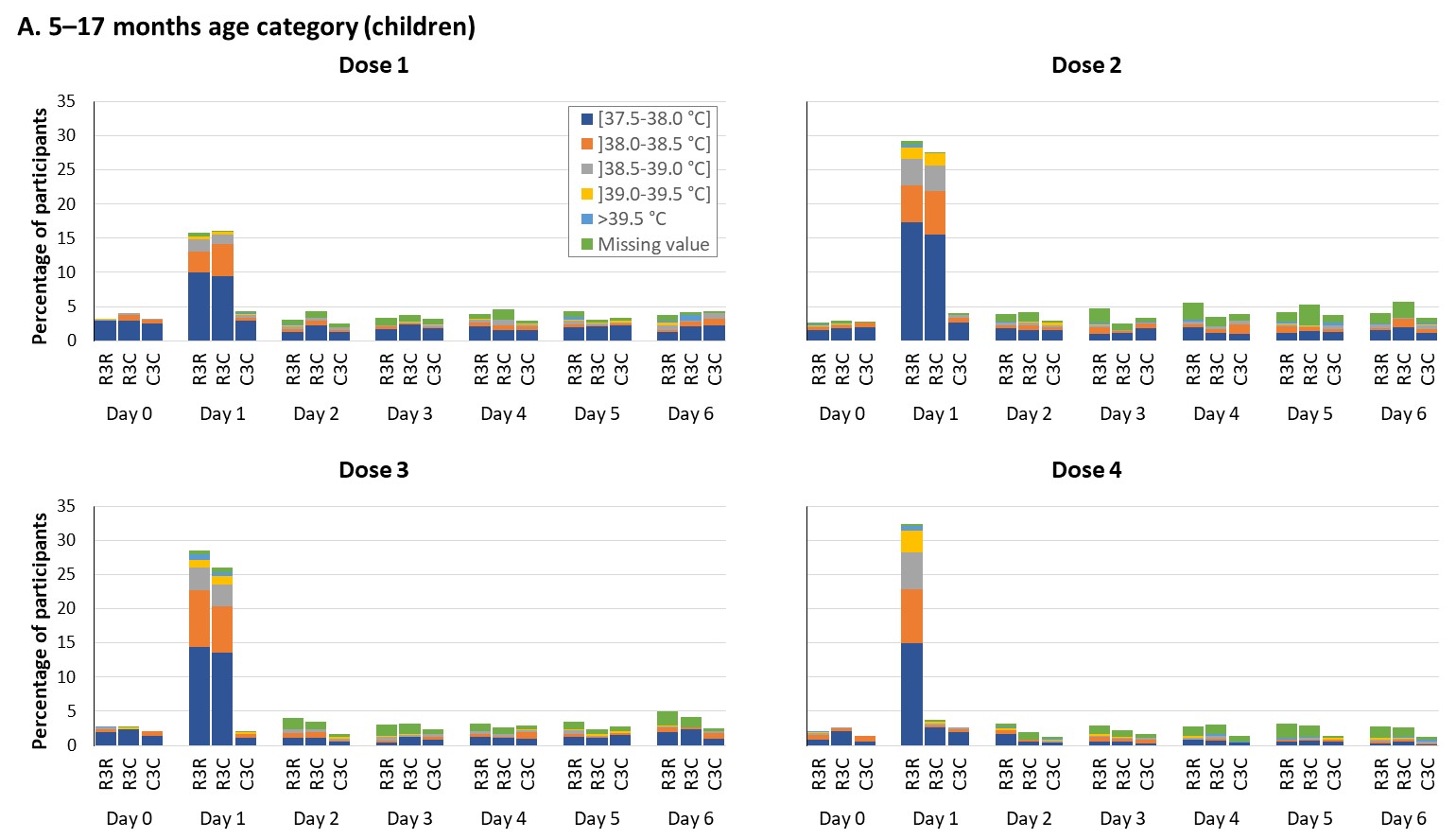
**

**
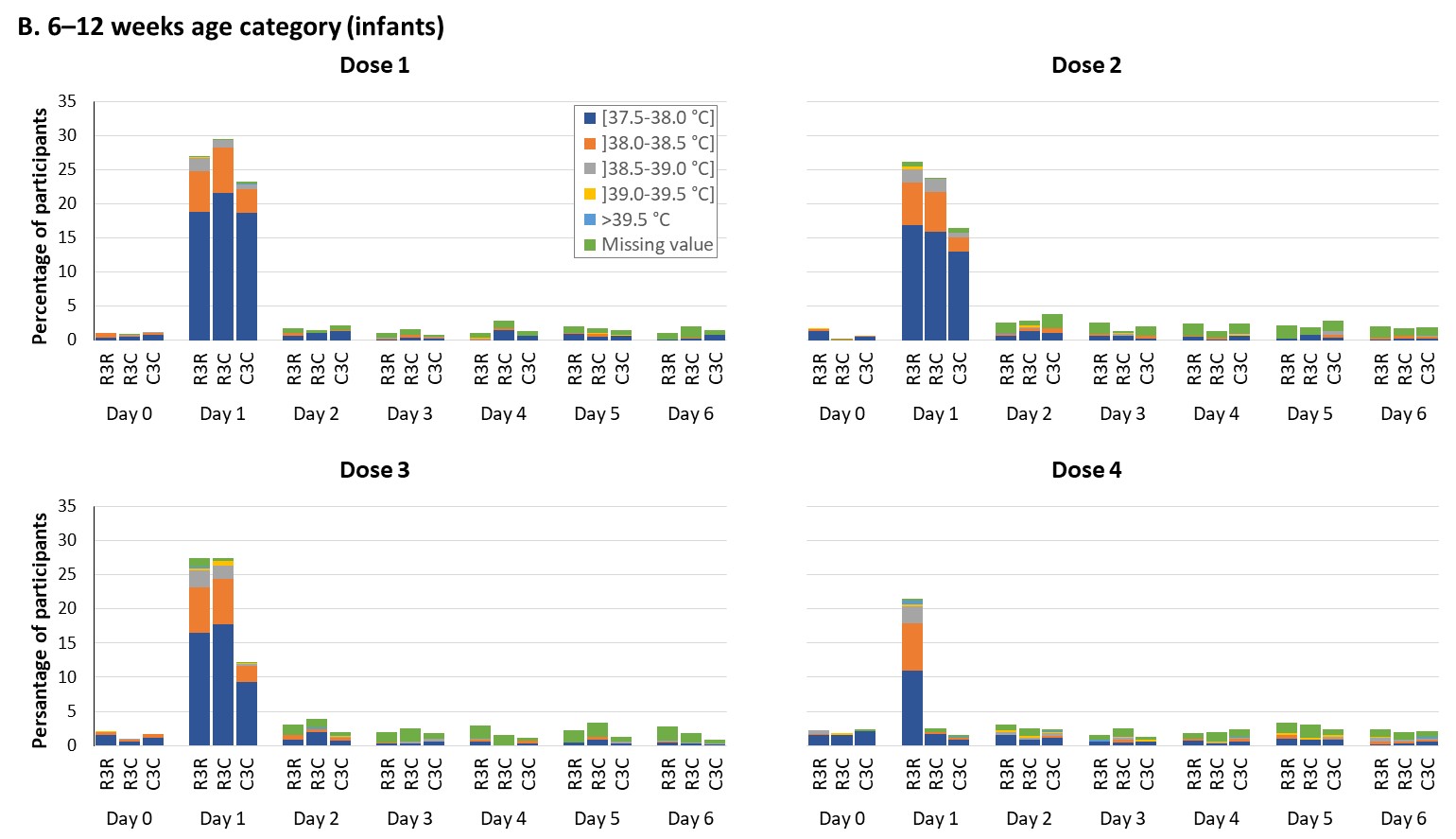
**

R3R, group receiving 4 doses of RTS,S/AS01; R3C, group receiving 3 doses of RTS,S/AS01 plus 1 dose of control vaccine; C3C, group receiving 4 doses of control vaccine.

**Figure S2. Distribution of general convulsive seizures (level 1–3 according to BCWG) within 7 days post-vaccination by dose in the 5–17 months age category**

| Days post | **dose 1** | | |  | Days post | **dose 1** | | |
| --- | --- | --- | --- | --- | --- | --- | --- | --- |
| vaccination | **R3R** | **R3C** | **C3C** |  | vaccination | **R3R** | **R3C** | **C3C** |
| Day 0 |  |  |  |  | Days 0–2 |  |  | 2 |
| Day 1 |  |  |  |  | Days 3–6 | 1 | 2 | 2 |
| Day 2 |  |  | 2 |  |  |  |  |  |
| Day 3 |  | 1 |  |  |  |  |  |  |
| Day 4 |  |  | 1 |  |  |  |  |  |
| Day 5 |  | 1 |  |  |  |  |  |  |
| Day 6 | 1 |  | 1 |  |  |  |  |  |
|  |  |  |  |  |  |  |  |  |
| Days post | **dose 2** | | |  | Days post | **dose 2** | | |
| vaccination | **R3R** | **R3C** | **C3C** |  | vaccination | **R3R** | **R3C** | **C3C** |
| Day 0 |  | 1 |  |  | Days 0–2 | 1 | 2 |  |
| Day 1 | 1 | 1 |  |  | Days 3–6 | 1 |  | 1 |
| Day 2 |  |  |  |  |  |  |  |  |
| Day 3 |  |  |  |  |  |  |  |  |
| Day 4 |  |  |  |  |  |  |  |  |
| Day 5 |  |  | 1 |  |  |  |  |  |
| Day 6 | 1 |  |  |  |  |  |  |  |
|  |  |  |  |  |  |  |  |  |
| Days post | **dose 3** | | |  | Days post | **dose 3** | | |
| vaccination | **R3R** | **R3C** | **C3C** |  | vaccination | **R3R** | **R3C** | **C3C** |
| Day 0 | 2 | 1 |  |  | Days 0–2 | 5 | 4 |  |
| Day 1 | 2 | 3 |  |  | Days 3–6 | 1 | 1 |  |
| Day 2 | 1 |  |  |  |  |  |  |  |
| Day 3 |  |  |  |  |  |  |  |  |
| Day 4 |  |  |  |  |  |  |  |  |
| Day 5 |  |  |  |  |  |  |  |  |
| Day 6 | 1 | 1 |  |  |  |  |  |  |
|  |  |  |  |  |  |  |  |  |
| Days post | **dose 4** | | |  | Days post | **dose 4** | | |
| vaccination | **R3R** | **R3C** | **C3C** |  | vaccination | **R3R** | **R3C** | **C3C** |
| Day 0 | 3 |  |  |  | Days 0–2 | 6 | 1 |  |
| Day 1 | 2 | 1 |  |  | Days 3–6 |  | 2 | 1 |
| Day 2 | 1 |  |  |  |  |  |  |  |
| Day 3 |  | 1 |  |  |  |  |  |  |
| Day 4 |  |  | 1 |  |  |  |  |  |
| Day 5 |  | 1 |  |  |  |  |  |  |
| Day 6 |  |  |  |  |  |  |  |  |

R3R, group receiving 4 doses of RTS,S/AS01; R3C, group receiving 3 doses of RTS,S/AS01 plus 1 dose of control vaccine; C3C, group receiving 4 doses of control vaccine. BCWG, Brighton Collaboration Working Group.

**Figure S3. Distribution of meningitis cases by age of onset in both age categories**

**
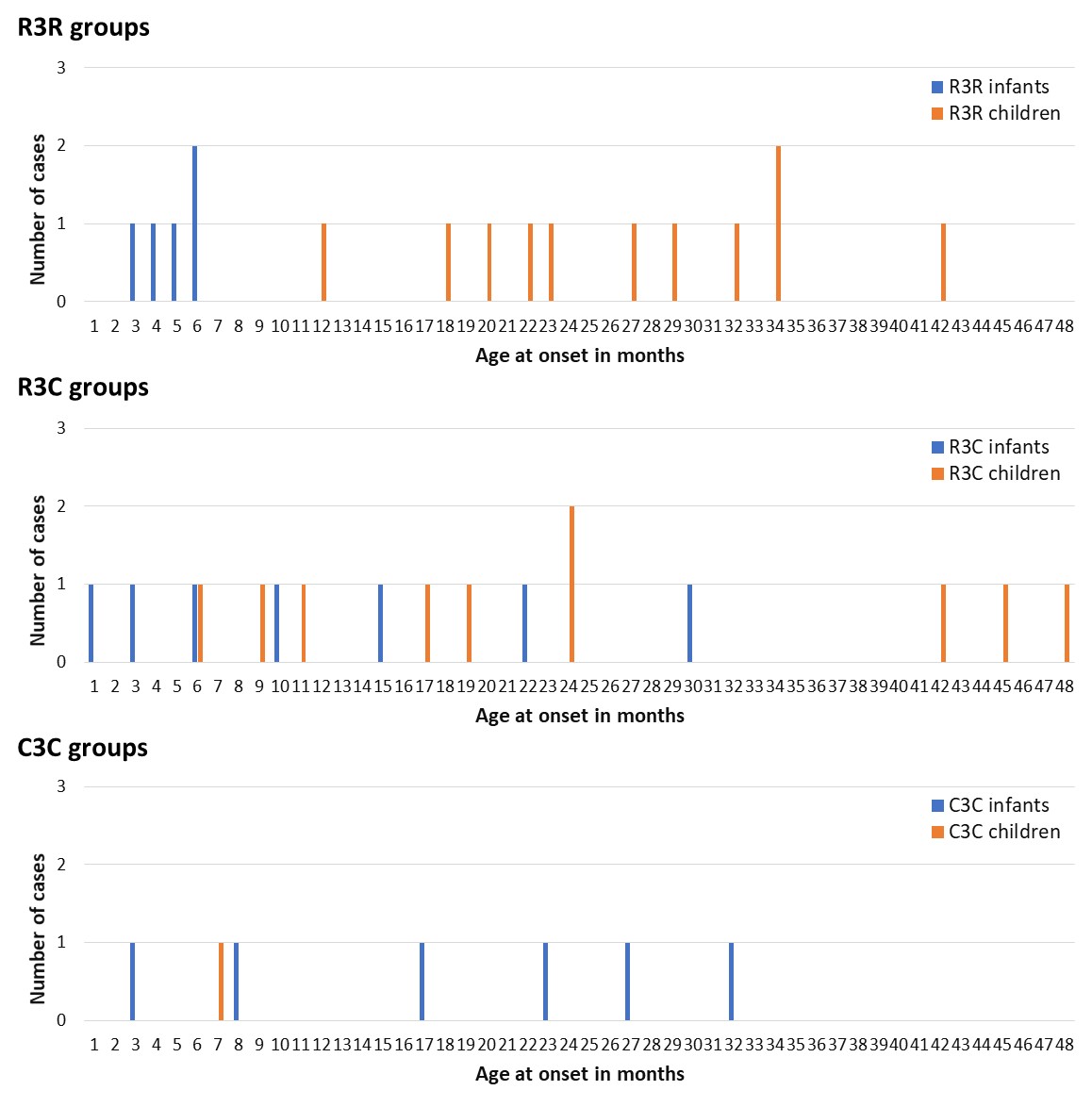
**

R3R, group receiving 4 doses of RTS,S/AS01; R3C, group receiving 3 doses of RTS,S/AS01 plus 1 dose of control vaccine; C3C, group receiving 4 doses of control vaccine.

**Figure S4. Distribution of meningitis cases in Lilongwe site (Malawi) over time and by etiology in both age categories**

**
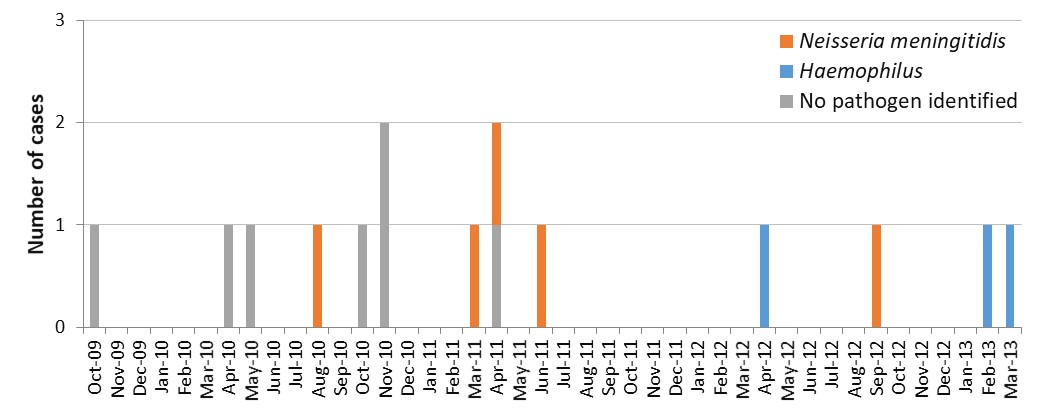
**
