# Supplementary material for: Safety profile of the RTS,S/AS01 malaria vaccine in infants and children: additional data from a phase III randomized controlled trial in sub-Saharan Africa
Source: Hum Vaccin Immunother. 2019 Apr 23;15(10):2386–98. doi: 10.1080/21645515.2019.1586040 (PMC6816384; doi:10.1080/21645515.2019.1586040)
Supplement: Supplemental Material [file khvi-15-10-1586040-s001.zip › Mal-055 safety mns_25JAN2019_clean_Supplement_Tables.docx]

**Table S1. Incidence (overall/dose) of solicited local and general symptoms in children aged 5–17 months and infants aged 6–12 weeks within 7 days after each of the first 3 doses of RTS,S/AS01 or control vaccine (ITT population)**

|  | | **5–17 months age category (children)** | | **6–12 weeks age category (infants)** | |
| --- | --- | --- | --- | --- | --- |
| **Symptom** | | **R3R+R3C**  N=4321 | **C3C**  N=2128 | **R3R+R3C**  N=4252 | **C3C**  N=2169 |
|  |  | % (95% CI) | % (95% CI) | % 95% CI | % (95% CI) |
| **Solicited local symptoms** | |  |  |  |  |
| Pain | all | 12.4 (11.4-13.4) | 5.8 (4.9-6.9) | 27.4 (26.0-28.7) | 25.2 (23.4-27.1) |
|  | Grade 3 | 0.1 (0.0-0.2) | 0.0 (0.0-0.2) | 0.5 (0.3-0.8) | 0.6 (0.3-1.0) |
| Redness | all | 3.1 (2.6-3.7) | 2.7 (2.0-3.5) | 9.7 (8.8-10.6) | 11.2 (9.9-12.6) |
|  | >20 mm | 0.2 (0.1-0.3) | 0.0 (0.0-0.2) | 0.2 (0.1-0.3) | 0.2 (0.1-0.5) |
| Swelling | all | 9.6 (8.7-10.5) | 7.6 (6.5-8.8) | 15.1 (14.0-16.2) | 16.8 (15.2-18.4) |
|  | >20 mm | 0.7 (0.5-1.0) | 0.0 (0.0-0.2) | 1.5 (1.2-1.9) | 2.7 (2.0-3.4) |
| **Solicited general symptoms** | | | | | |
| Drowsiness | all | 6.6 (5.9-7.4) | 4.4 (3.5-5.3) | 9.9 (9.1-10.9) | 7.6 (6.5-8.8) |
|  | Grade 3 | 0.1 (0.0-0.3) | 0.0 (0.0-0.2) | 0.0 (0.0-0.2) | 0.1 (0.0-0.3) |
|  | Related | 3.4 (2.9-4.0) | 2.3 (1.7-3.1) | 5.0 (4.3-5.7) | 2.8 (2.1-3.5) |
|  | Grade 3 related | 0.0 (0.0-0.2) | 0.0 (0.0-0.2) | 0.0 (0.0-0.1) | 0.0 (0.0-0.2) |
| Irritability | all | 11.5 (10.5-12.4) | 5.3 (4.4-6.3) | 22.2 (21.0-23.5) | 17.7 (16.1-19.4) |
|  | Grade 3 | 0.1 (0.0-0.2) | 0.0 (0.0-0.2) | 0.5 (0.3-0.7) | 0.2 (0.1-0.5) |
|  | Related | 5.9 (5.2-6.6) | 2.8 (2.1-3.6) | 12.8 (11.8-13.9) | 9.0 (7.8-10.3) |
|  | Grade 3 related | 0.0 (0.0-0.1) | 0.0 (0.0-0.2) | 0.3 (0.2-0.5) | 0.1 (0.0-0.4) |
| Loss of appetite | all | 11.4 (10.4-12.3) | 7.4 (6.3-8.6) | 7.9 (7.1-8.7) | 6.5 (5.5-7.6) |
|  | Grade 3 | 0.1 (0.0-0.3) | 0.0 (0.0-0.2) | 0.0 (0.0-0.2) | 0.0 (0.0-0.3) |
|  | Related | 5.5 (4.8-6.2) | 3.1 (2.4-3.9) | 4.0 (3.5-4.7) | 2.4 (1.8-3.1) |
|  | Grade 3 related | 0.0 (0.0-0.1) | 0.0 (0.0-0.2) | 0.0 (0.0-0.2) | 0.0 (0.0-0.2) |
| Fever | All (≥37.5°C) | 31.1 (29.7-32.5) | 13.4 (12.0-14.9) | 30.6 (29.2-32.0) | 21.1 (19.4-22.8) |
|  | >39.0°C | 2.5 (2.1-3.1) | 1.1 (0.7-1.7) | 0.6 (0.4-0.9) | 0.5 (0.3-0.9) |
|  | ≥37.5°C related | 16.9 (15.8-18.0) | 5.9 (5.0-7.0) | 20.8 (19.6-22.0) | 12.6 (11.2-14.1) |
|  | >39.0°C related | 1.5 (1.1-1.9) | 0.3 (0.1-0.7) | 0.4 (0.2-0.6) | 0.3 (0.1-0.7) |

ITT, intention to treat; R3R+R3C, groups receiving 4 doses of RTS,S/AS01 or 3 doses of RTS,S/AS01 plus 1 dose of control vaccine; C3C, group receiving 4 doses of control vaccine; N, number of administered doses; %, percentage of doses followed by at least one type of symptom; 95% CI, exact 95% confidence interval. Grade 3 symptoms were defined as crying when the limb was moved or limb was spontaneously painful (pain), crying that could not be comforted or prevented normal activity (irritability), preventing normal activity (drowsiness) and not eating at all (loss of appetite).

**Table S2. Incidence of solicited local and general symptoms in children aged 5–17 months and infants aged 6–12 weeks within 7 days after a fourth dose of RTS,S/AS01 or control vaccine (ITT population)**

|  | | **5–17 months age category (children)** | | | **6–12 weeks age category (infants)** | | |
| --- | --- | --- | --- | --- | --- | --- | --- |
| **Symptom** | | **R3R**  N=641 | **R3C**  N=639 | **C3C**  N=633 | **R3R**  N=608 | **R3C**  N=625 | **C3C**  N=621 |
|  |  | **% (95% CI)** | **% (95% CI)** | **% (95% CI)** | **% (95% CI)** | **% (95% CI)** | **% (95% CI)** |
| **Solicited local symptoms** | |  |  |  |  |  |  |
| Pain | all | 17.0 (14.2-20.1) | 7.0 (5.2-9.3) | 6.5 (4.7-8.7) | 9.7 (7.5-12.3) | 4.6 (3.1-6.6) | 4.0 (2.6-5.9) |
|  | Grade 3 | 0.0 (0.0-0.6) | 0.0 (0.0-0.6) | 0.0 (0.0-0.6) | 0.0 (0.0-0.6) | 0.0 (0.0-0.6) | 0.0 (0.0-0.6) |
| Redness | all | 2.3 (1.3-3.8) | 2.0 (1.1-3.5) | 1.3 (0.5-2.5) | 1.5 (0.7-2.8) | 1.9 (1.0-3.3) | 1.4 (0.7-2.7) |
|  | >20 mm | 0.5 (0.1-1.4) | 0.0 (0.0-0.6) | 0.0 (0.0-0.6)  4.7 (3.2-6.7) | 0.2 (0.0-0.9) | 0.0 (0.0-0.6) | 0.0 (0.0-0.6) |
| Swelling | all | 6.6 (4.8-8.8) | 5.5 (3.8-7.5) |  | 7.4 (5.4-9.8) | 4.5 (3.0-6.4) | 6.9 (5.1-9.2) |
|  | >20 mm | 1.4 (0.6-2.6) | 0.2 (0.0-0.9) | 0.0 (0.0-0.6) | 0.8 (0.3-1.9) | 0.0 (0.0-0.6) | 0.3 (0.0-1.2) |
| **Solicited general symptoms** | | | | | | | |
| Drowsiness | all | 8.6 (6.5-11.0) | 3.4 (2.2-5.2) | 3.3 (2.1-5.0) | 5.4 (3.8-7.5) | 3.0 (1.8-4.7) | 2.4 (1.4-4.0) |
|  | Grade 3 | 0.2 (0.0-0.9) | 0.0 (0.0-0.6) | 0.0 (0.0-0.6) | 0.0 (0.0-0.6) | 0.0 (0.0-0.6) | 0.0 (0.0-0.6) |
|  | Related | 5.3 (3.7-7.3) | 1.6 (0.8-2.9) | 2.1 (1.1-3.5) | 3.1 (1.9-4.8 | 1.0 (0.4-2.1) | 0.8 (0.3-1.9) |
|  | Grade 3 related | 0.0 (0.0-0.6) | 0.0 (0.0-0.6) | 0.0 (0.0-0.6) | 0.0 (0.0-0.6) | 0.0 (0.0-0.6) | 0.0 (0.0-0.6) |
| Irritability | all | 9.8 (7.6-12.4) | 3.9 (2.5-5.7) | 2.8 (1.7-4.5) | 7.6 (5.6-10.0) | 3.7 (2.3-5.5) | 3.7 (2.4-5.5) |
|  | Grade 3 | 0.2 (0.0-0.9) | 0.0 (0.0-0.6) | 0.0 (0.0-0.6) | 0.0 (0.0-0.6) | 0.0 (0.0-0.6) | 0.0 (0.0-0.6) |
|  | Related | 6.2 (4.5-8.4) | 1.9 (1.0-3.3) | 1.3 (0.5-2.5) | 4.4 (2.9-6.4) | 1.6 (0.8-2.9) | 1.0 (0.4-2.1) |
|  | Grade 3 related | 0.2 (0.0-0.9) | 0.0 (0.0-0.6) | 0.0 (0.0-0.6) | 0.0 (0.0-0.6) | 0.0 (0.0-0.6) | 0.0 (0.0-0.6) |
| Loss of appetite | all | 10.3 (8.1-12.9) | 4.2 (2.8-6.1) | 3.3 (2.1-5.0) | 7.4 (5.4-9.8) | 4.3 (2.9-6.2) | 2.9 (1.7-4.5) |
|  | Grade 3 | 0.2 (0.0-0.9) | 0.0 (0.0-0.6) | 0.0 (0.0-0.6) | 0.0 (0.0-0.6) | 0.0 (0.0-0.6) | 0.0 (0.0-0.6) |
|  | Related | 6.1 (4.4-8.2) | 2.2 (1.2-3.6) | 2.1 (1.1-3.5) | 4.3 (2.8-6.2) | 1.3 (0.6-2.5) | 1.0 (0.4-2.1) |
|  | Grade 3 related | 0.2 (0.0-0.9) | 0.0 (0.0-0.6) | 0.0 (0.0-0.6) | 0.0 (0.0-0.6) | 0.0 (0.0-0.6) | 0.0 (0.0-0.6) |
| Fever | All (≥37.5°C) | 36.3 (32.6-40.2) | 11.0 (8.6-13.6) | 7.1 (5.2-9.4) | 25.0 (21.6-28.6) | 8.3 (6.3-10.8) | 9.3 (7.2-11.9) |
|  | >39.0°C | 5.3 (3.7-7.3) | 0.9 (0.3-2.0) | 0.8 (0.3-1.8) | 1.5 (0.7-2.8) | 1.1 (0.5-2.3) | 1.6 (0.8-2.9) |
|  | ≥37.5°C related | 23.6 (20.3-27.0) | 4.5 (3.1-6.5) | 2.5 (1.5-4.1) | 13.2 (10.6-16.1) | 2.4 (1.3-3.9) | 2.9 (1.7-4.5) |
|  | >39.0°C related | 3.7 (2.4-5.5) | 0.2 (0.0-0.9) | 0.0 (0.0-0.6) | 0.8 (0.3-1.9) | 0.2 (0.0-0.9) | 0.5 (0.1-1.4) |

ITT, intention to treat; R3R, group receiving 4 doses of RTS,S/AS01; R3C, group receiving 3 doses of RTS,S/AS01 plus 1 dose of control vaccine; C3C, group receiving 4 doses of control vaccine; N, number of children/infants with the administered dose; %, percentage of children/infants reporting the symptom at least once; 95% CI, exact 95% confidence interval. Grade 3 symptoms were defined as crying when the limb was moved or limb was spontaneously painful (pain), crying that could not be comforted or prevented normal activity (irritability), preventing normal activity (drowsiness) and not eating at all (loss of appetite).

**Table S3. Gestational age of the infants born prematurely**

|  | | **R3R+R3C  N=244** | **C3C  N=118** |
| --- | --- | --- | --- |
|  | | **n (%)** | **n (%)** |
| Gestational age (weeks) | 27 | 1 (0.4) | 0 (0.0) |
|  | 28 | 4 (1.6) | 0 (0.0) |
|  | 29 | 2 (0.8) | 0 (0.0) |
|  | 30 | 2 (0.8) | 2 (1.7) |
|  | 32 | 14 (5.7) | 11 (9.3) |
|  | 33 | 4 (1.6) | 0 (0.0) |
|  | 34 | 27 (11.1) | 12 (10.2) |
|  | 35 | 7 (2.9) | 6 (5.1) |
|  | 36 | 183 (75.0) | 87 (73.7) |
| R3R+R3C, groups receiving 4 doses of RTS,S/AS01 or 3 doses of RTS,S/AS01 plus 1 dose of control vaccine; C3C, group receiving 4 doses of control vaccine; N, total number of premature infants per group; n (%), number (percentage) of infants with the specified gestational age | | | |

**Table S4. Predefined list of potential immune-mediated disorders**

| **Gastrointestinal disorders** | **Metabolic diseases** | **Skin disorders** |
| --- | --- | --- |
| Crohn’s disease  Ulcerative colitis  Ulcerative proctitis  Celiac disease | Autoimmune thyroiditis  Grave's or Basedow’s disease  Hashimoto thyroiditis  Diabetes Mellitus Type 1  Addison’s disease | Psoriasis  Vitiligo  Raynaud’s phenomenon  Erythema nodosum  Autoimmune bullous skin diseases |
| **Neuroinflammatory disorders** | **Musculoskeletal disorders** | **Others** |
| Optic neuritis  Multiple sclerosis  Demyelinating disease  Transverse myelitis  Guillain-Barré syndrome  Myasthenia gravis  Encephalitis  Neuritis  Bell’s palsy | Systemic lupus erythematosus  Cutaneous lupus  Sjogren’s syndrome  Scleroderma  Dermatomyositis  Polymyositis  Rheumatoid arthritis  Juvenile arthritis  Polymyalgia rheumatic  Reactive arthritis  Psoriatic arthropathy  Ankylosing spondylitis  Undifferentiated spondyloarthropathy | Auto-immune hemolytic anemia  Idiopathic thrombocytopenic purpura  Antiphospholipid syndrome  Vasculitis  Pernicious anemia  Autoimmune hepatitis  Primary biliary cirrhosis  Primary sclerosing cholangitis  Autoimmune glomerulonephritis  Autoimmune uveitis  Autoimmune myocarditis  Sarcoidosis  Stevens-Johnson syndrome |

**Table S5. Multivariate Cox regression analysis in boys and girls to model risk factors known to be associated with mortality**

| **Variable** | **Comparison** | **Hazard Ratio** | **p-value** |
| --- | --- | --- | --- |
| **Boys** |  |  |  |
| RTS,S/AS01 vaccination | 3 or 4 doses vs control | 0.82 | 0.2328 |
| Age category | 5–17 months vs 6–12 weeks | 0.57 | 0.0013 |
| Site (vs Agogo) | Bagamoyo | 3.94 | 0.0163 |
|  | Kilifi | 1.79 | 0.4268 |
|  | Kintampo | 3.25 | 0.0489 |
|  | Kombewa | 4.80 | 0.0053 |
|  | Korogwe | 2.69 | 0.1109 |
|  | Lambarene | 3.99 | 0.0266 |
|  | Lilongwe | 2.69 | 0.0993 |
|  | Manhica | 8.11 | 0.0002 |
|  | Nanoro | 1.81 | 0.3557 |
|  | Siaya | 3.40 | 0.0302 |
| HIV status^a^ | Confirmed positive vs negative/unknown | 14.92 | 0.0001 |
| Baseline anemia | Hb <8 g/dL | 1.44 | 0.2273 |
| Distance from inpatient facility | >5 km | 0.84 | 0.4401 |
| Baseline weight-for-age | <-2 z-scores | 2.04 | 0.0004 |
| **Girls** |  |  |  |
| RTS,S/AS01 vaccination | 3 or 4 doses vs control | 1.86 | 0.0015 |
| Age category | 5–17 months vs 6–12 weeks | 0.65 | 0.0126 |
| Site (vs Agogo) | Bagamoyo | 1.64 | 0.2887 |
|  | Kilifi | 0.53 | 0.3754 |
|  | Kintampo | 1.55 | 0.3684 |
|  | Kombewa | 1.74 | 0.2243 |
|  | Korogwe | 1.89 | 0.1895 |
|  | Lambarene | 1.44 | 0.5223 |
|  | Lilongwe | 0.96 | 0.9345 |
|  | Manhica | 2.76 | 0.0232 |
|  | Nanoro | 1.24 | 0.6683 |
|  | Siaya | 1.25 | 0.634 |
| HIV status | Confirmed positive vs negative/unknown | 15.35 | 0.0001 |
| Baseline anemia | Hb <8 g/dL | 1.95 | 0.0223 |
| Distance from inpatient facility | >5 km | 1.31 | 0.2581 |
| Baseline weight-for-age | <-2 z-scores | 2.38 | 0.0001 |

Hb, hemoglobin. ^a^ HIV testing was not a study procedure and was not systematically performed at all study centers. Confirmed positive cases included all children and infants identified to be HIV-infected (stage I/II) at enrollment or subsequently diagnosed on clinical suspicion, and confirmed to be HIV positive by polymerase chain reaction or by antibody testing at an age of 18 months or older. Negative/unknown were children and infants not confirmed as HIV-positive and therefore included both HIV-negative (some confirmed, most not confirmed as negative) and HIV-positive (but not known to be positive) participants.

**Table S6. Fatal serious adverse events by MedDRA primary system organ class and preferred term and gender for both age categories combined (ITT population)**

| **Primary System Organ Class** | **Preferred Term** | **C3C** | | | | **R3R+R3C** | | | |
| --- | --- | --- | --- | --- | --- | --- | --- | --- | --- |
|  |  | **Girls** | | **Boys** | | **Girls** | | **Boys** | |
|  |  | **N=2603** | | **N=2550** | | **N=5091** | | **N=5215** | |
|  |  | **n** | **%** | **n** | **%** | **n** | **%** | **n** | **%** |
| **Total number of subjects with fatal SAEs** | | 33 | 1.27 | 55 | 2.16 | 123 | 2.42 | 95 | 1.82 |
| **Total number of events with fatal outcome^a^** | | 65 | 2.50 | 120 | 4.71 | 228 | 4.48 | 182 | 3.49 |
| **Blood and lymphatic system disorders** | Anemia | 5 | 0.19 | 11 | 0.43 | 16 | 0.31 | 18 | 0.35 |
|  | Disseminated intravascular coagulation | 0 |  | 0 |  | 0 |  | 1 | 0.02 |
|  | Hemolytic anemia | 1 | 0.04 | 0 |  | 0 |  | 1 | 0.02 |
|  | Neutropenia | 0 |  | 0 |  | 1 | 0.02 | 1 | 0.02 |
| **Congenital, familial and genetic disorders** | Atrial septal defect | 0 |  | 0 |  | 1 | 0.02 | 0 |  |
|  | Cerebral palsy | 0 |  | 0 |  | 1 | 0.02 | 0 |  |
|  | Congenital megacolon | 0 |  | 0 |  | 0 |  | 1 | 0.02 |
|  | Fallot’s tetralogy | 0 |  | 0 |  | 1 | 0.02 | 0 |  |
|  | Sickle cell anemia | 0 |  | 0 |  | 0 |  | 1 | 0.02 |
|  | Sickle cell anemia with crisis | 0 |  | 1 | 0.04 | 0 |  | 1 | 0.02 |
| **Gastrointestinal disorders** | Enteritis | 1 | 0.04 | 2 | 0.08 | 1 | 0.02 | 0 |  |
|  | Hematemesis | 0 |  | 0 |  | 0 |  | 1 | 0.02 |
|  | Intestinal obstruction | 0 |  | 0 |  | 0 |  | 1 | 0.02 |
|  | Intestinal perforation | 0 |  | 0 |  | 0 |  | 1 | 0.02 |
|  | Intussusception | 0 |  | 0 |  | 0 |  | 1 | 0.02 |
| **General disorders and administration site conditions** | Death^b^ | 1 | 0.04 | 2 | 0.08 | 5 | 0.10 | 2 | 0.04 |
|  | Drowning | 2 | 0.08 | 2 | 0.08 | 2 | 0.04 | 4 | 0.08 |
|  | Hypothermia | 0 |  | 0 |  | 1 | 0.02 | 0 |  |
|  | Pyrexia | 1 | 0.04 | 4 | 0.16 | 3 | 0.06 | 1 | 0.02 |
| **Infections and infestations** | Atypical pneumonia | 0 |  | 0 |  | 1 | 0.02 | 0 |  |
|  | Bronchitis | 0 |  | 2 | 0.08 | 0 |  | 0 |  |
|  | Bronchopneumonia | 2 | 0.08 | 1 | 0.04 | 7 | 0.14 | 3 | 0.06 |
|  | Burn infection | 0 |  | 0 |  | 0 |  | 1 | 0.02 |
|  | Cerebral malaria | 0 |  | 0 |  | 5 | 0.10 | 2 | 0.04 |
|  | Disseminated tuberculosis | 0 |  | 1 | 0.04 | 0 |  | 0 |  |
|  | Dysentery | 0 |  | 0 |  | 2 | 0.04 | 1 | 0.02 |
|  | Encephalitis | 0 |  | 2 | 0.08 | 2 | 0.04 | 1 | 0.02 |
|  | Encephalitis viral | 0 |  | 0 |  | 0 |  | 1 | 0.02 |
|  | Febrile infection | 0 |  | 0 |  | 0 |  | 1 | 0.02 |
|  | Gastroenteritis | 6 | 0.23 | 12 | 0.47 | 25 | 0.49 | 21 | 0.40 |
|  | Gastroenteritis shigella | 0 |  | 0 |  | 1 | 0.02 | 0 |  |
|  | HIV associated nephropathy | 0 |  | 1 | 0.04 | 0 |  | 0 |  |
|  | HIV infection | 5 | 0.19 | 5 | 0.20 | 13 | 0.26 | 7 | 0.13 |
|  | HIV infection WHO clinical stage III | 0 |  | 0 |  | 1 | 0.02 | 0 |  |
|  | HIV infection WHO clinical stage IV | 0 |  | 0 |  | 1 | 0.02 | 0 |  |
|  | Klebsiella sepsis | 0 |  | 0 |  | 0 |  | 1 | 0.02 |
|  | Lobar pneumonia | 1 | 0.04 | 2 | 0.08 | 0 |  | 0 |  |
|  | Lymph node tuberculosis | 0 |  | 1 | 0.04 | 0 |  | 0 |  |
|  | Malaria | 7 | 0.27 | 8 | 0.31 | 19 | 0.37 | 19 | 0.36 |
|  | Measles | 0 |  | 0 |  | 1 | 0.02 | 0 |  |
|  | Meningitis | 1 | 0.04 | 1 | 0.04 | 4 | 0.08 | 0 |  |
|  | Meningitis pneumococcal | 0 |  | 2 | 0.08 | 1 | 0.02 | 2 | 0.04 |
|  | Oral candidiasis | 1 | 0.04 | 1 | 0.04 | 0 |  | 0 |  |
|  | Oropharyngeal candidiasis | 0 |  | 0 |  | 0 |  | 1 | 0.02 |
|  | Otitis media | 0 |  | 1 | 0.04 | 0 |  | 2 | 0.04 |
|  | Pneumococcal sepsis | 1 | 0.04 | 2 | 0.08 | 2 | 0.04 | 2 | 0.04 |
|  | Pneumocystis jirovecii pneumonia | 1 | 0.04 | 0 |  | 2 | 0.04 | 2 | 0.04 |
|  | Pneumonia | 5 | 0.19 | 12 | 0.47 | 26 | 0.51 | 23 | 0.44 |
|  | Pulmonary tuberculosis | 0 |  | 0 |  | 1 | 0.02 | 1 | 0.02 |
|  | Rabies | 0 |  | 0 |  | 0 |  | 1 | 0.02 |
|  | Respiratory tract infection | 0 |  | 1 | 0.04 | 0 |  | 1 | 0.02 |
|  | Salmonella sepsis | 1 | 0.04 | 3 | 0.12 | 3 | 0.06 | 2 | 0.04 |
|  | Sepsis | 4 | 0.15 | 6 | 0.24 | 14 | 0.27 | 8 | 0.15 |
|  | Septic shock | 0 |  | 0 |  | 0 |  | 1 | 0.02 |
|  | Skin bacterial infection | 0 |  | 0 |  | 1 | 0.02 | 0 |  |
|  | Staphylococcal sepsis | 0 |  | 0 |  | 1 | 0.02 | 0 |  |
|  | Streptococcal sepsis | 0 |  | 1 | 0.04 | 1 | 0.02 | 1 | 0.02 |
|  | Toxic shock syndrome | 0 |  | 0 |  | 0 |  | 1 | 0.02 |
|  | Tuberculosis | 1 | 0.04 | 2 | 0.08 | 0 |  | 1 | 0.02 |
|  | Typhoid fever | 0 |  | 1 | 0.04 | 0 |  | 0 |  |
|  | Upper respiratory tract infection | 1 | 0.04 | 0 |  | 0 |  | 0 |  |
|  | Urinary tract infection | 0 |  | 2 | 0.08 | 0 |  | 1 | 0.02 |
|  | Varicella | 0 |  | 0 |  | 1 | 0.02 | 0 |  |
| **Injury, poisoning and procedural complications** | Burns second degree | 0 |  | 0 |  | 1 | 0.02 | 0 |  |
|  | Crush injury | 0 |  | 0 |  | 1 | 0.02 | 0 |  |
|  | Dislocation of vertebra | 0 |  | 0 |  | 0 |  | 1 | 0.02 |
|  | Head injury | 0 |  | 0 |  | 2 | 0.04 | 1 | 0.02 |
|  | Herbal toxicity | 1 | 0.04 | 1 | 0.04 | 4 | 0.08 | 2 | 0.04 |
|  | Road traffic accident | 0 |  | 1 | 0.04 | 1 | 0.02 | 0 |  |
|  | Thermal burn | 1 | 0.04 | 0 |  | 1 | 0.02 | 2 | 0.04 |
| **Metabolism and nutrition disorders** | Hypoglycemia | 2 | 0.08 | 0 |  | 4 | 0.08 | 2 | 0.04 |
|  | Kwashiorkor | 0 |  | 1 | 0.04 | 4 | 0.08 | 0 |  |
|  | Malnutrition | 1 | 0.04 | 7 | 0.27 | 8 | 0.16 | 5 | 0.10 |
|  | Marasmus | 3 | 0.12 | 1 | 0.04 | 8 | 0.16 | 3 | 0.06 |
| **Neoplasms benign, malignant and unspecified (incl cysts and polyps)** | Acute promyelocytic leukemia | 0 |  | 0 |  | 1 | 0.02 | 0 |  |
|  | Brain neoplasm | 0 |  | 0 |  | 1 | 0.02 | 0 |  |
| **Nervous system disorders** | Convulsion | 4 | 0.15 | 10 | 0.39 | 11 | 0.22 | 13 | 0.25 |
|  | Encephalopathy | 0 |  | 0 |  | 0 |  | 1 | 0.02 |
|  | Febrile convulsion | 2 | 0.08 | 4 | 0.16 | 4 | 0.08 | 6 | 0.12 |
|  | Hemorrhage intracranial | 0 |  | 0 |  | 1 | 0.02 | 0 |  |
|  | Loss of consciousness | 0 |  | 0 |  | 1 | 0.02 | 0 |  |
|  | Uremic encephalopathy | 0 |  | 1 | 0.04 | 0 |  | 0 |  |
| **Renal and urinary disorders** | Glomerulonephritis acute | 0 |  | 0 |  | 1 | 0.02 | 0 |  |
|  | Renal tubular necrosis | 0 |  | 0 |  | 0 |  | 1 | 0.02 |
| **Respiratory, thoracic and mediastinal disorders** | Asphyxia | 0 |  | 1 | 0.04 | 1 | 0.02 | 0 |  |
|  | Aspiration | 0 |  | 0 |  | 0 |  | 1 | 0.02 |
|  | Obstructive airways disorder | 0 |  | 0 |  | 1 | 0.02 | 0 |  |
|  | Pneumonia aspiration | 1 | 0.04 | 0 |  | 4 | 0.08 | 1 | 0.02 |
|  | Pulmonary edema | 0 |  | 0 |  | 1 | 0.02 | 0 |  |
| **Skin and subcutaneous tissue disorders** | Skin lesion | 1 | 0.04 | 0 |  | 0 |  | 0 |  |
| **Vascular disorders** | Shock | 1 | 0.04 | 1 | 0.04 | 0 |  | 0 |  |

^a^More than one event could be reported per participant. ^b^ (Suspected) cause of death could not be determined. MedDRA, Medical Dictionary for Regulatory Activities; ITT, intention to treat; R3R, group receiving 4 doses of RTS,S/AS01; R3C, group receiving 3 doses of RTS,S/AS01 plus 1 dose of control vaccine; C3C, group receiving 4 doses of control vaccine; N, total number of children/infants per group; n (%), number (percentage) of fatal SAEs in a given category, or, for “Total number of subjects with fatal SAEs”, number (percentage) of subjects with fatal SAE.
