# Supplementary material for: Safety profile of the RTS,S/AS01 malaria vaccine in infants and children: additional data from a phase III randomized controlled trial in sub-Saharan Africa
Source: Hum Vaccin Immunother. 2019 Apr 23;15(10):2386–98. doi: 10.1080/21645515.2019.1586040 (PMC6816384; doi:10.1080/21645515.2019.1586040)
Supplement: Supplemental Material [file khvi-15-10-1586040-s001.zip › Mal-055 safety mns_25JAN2019_clean_Supplement_TEXT.docx]

**Supplemental online material**

**Supplementary methods**

***Study design***

This study, conducted between March 2009 and January 2014, was the first phase III malaria vaccine trial, initiated in collaboration with the Clinical Trial Partnership Committee (CTPC, representing several leading research institutes and academic partners from Africa, the European Union, and the United States [US]), and in consultation with African, European and US regulatory authorities. World Health Organization (WHO) recommendations were also incorporated in the study design.

Parents or legally authorized representatives of all participants provided written or thumb-printed and witnessed informed consent at enrollment in the primary study.

The trial protocol was approved by the ethical review board at each study center and partner institution and by the national regulatory authority in each country and the trial was undertaken in accordance with the provisions of the Good Clinical Practice guidelines.

***Study vaccines***

The AS01_E_ Adjuvant System contains 25 µg 3-O-desacyl-4’- monophosphoryl lipid A, 25 µg *Quillaja saponaria Molina* fraction 21 (Antigenics Inc., a wholly owned subsidiary of Agenus Inc., a Delaware, US corporation) and liposome.

***Safety oversight***

The trial was overseen by an Independent Data Monitoring Committee, who reviewed all serious adverse events (SAEs), all withdrawals due to adverse events (AEs), and any information concerning events that might adversely affect participants’ safety or conduct of the study. To ensure collection of quality data across all centers, a large capacity-building initiative was put in place in terms of staff training, increased standard of patient care, and upgrade of laboratories and other facilities. Standardized laboratory methods and quality assurance processes were implemented at each site before study start.^1^ Access to insecticide-treated bednets was optimized for all screened children; net use and condition were assessed during protocol-specified home visits.

***Algorithm for the evaluation of hospital admission as a potential case of severe malaria***

For all acute hospital admissions (i.e., except planned admissions for medical investigation/care or elective surgery and trauma admissions) the following markers of severe disease were collected:

- malaria parasite density
- blood culture
- hemoglobin
- blood glucose, lactate, and base excess determination

Lumbar puncture was indicated by the presence of:

- seizure except simple febrile seizure (defined as associated with fever, lasting for 5 minutes or less, generalized as opposed to focal, not followed by transient or persistent neurological abnormalities, occurring in a child ≥6 months of age, with full recovery within 1 hour)
- Blantyre Coma Score (BCS) <5 (children ≤9 months of age <4 [in association with best motor response of 1])
- prostration in child <3 years of age
- meningism/stiff neck/bulging fontanelle
- clinician’s judgment

Cerebrospinal fluid samples from participants with suspicion of meningitis were tested for Salmonella species, *Neisseria meningitidis*, *Haemophilus influenzae*, *Streptococcus pneumoniae* as per protocol.

After the imbalance of meningitis cases between RTS,S/AS01 and control groups was first detected, polymerase chain reaction testing was to be performed on cerebrospinal fluid samples, when available, for the following pathogens: Epstein-Barr virus, cytomegalovirus, herpes simplex virus, and enterovirus; a routine test for Cryptococcus was also performed.

Chest X-ray was indicated by the presence of:

- tachypnea (≥50 breaths per minute in a child <1 year and ≥40 breaths per minute in a child ≥1 year)
- lower chest wall indrawing
- abnormally deep breathing
- clinician’s judgment

The primary case definition of clinical malaria included an illness in a child brought to a study facility with an axillary temperature of 37.5°C or more and *Plasmodium falciparum* asexual parasitemia (>5,000 parasites per µL) or a case of malaria meeting the primary case definition of severe malaria.

The primary case definition of severe malaria included *P. falciparum* >5,000 parasites per µL, with one or more markers of disease severity among:

- prostration
- respiratory distress
- Blantyre coma score (BSC) ≤2
- seizures 2 or more
- hypoglycemia <2.2 mmol/L
- acidosis base excess ≤-10.0 mmol/L
- lactate ≥5.0 mmol/L
- anemia <5.0 g/dL

and without the following co-morbidities:

- radiographically proven pneumonia
- meningitis on cerebrospinal fluid examination
- positive blood culture
- gastroenteritis with dehydration

The secondary case definition 1 of severe malaria was the same as the primary case definition, but did not require the exclusion of co-morbidities.

***Determination of cause of death***

At the end of the study and extension phase, all deaths were reviewed by a central panel composed of 3 experienced verbal autopsy reviewers using all available information (SAE forms, verbal autopsy forms, information on meningitis cases). Each panel member independently reviewed each death. The reviewers recorded: 1) the disease(s) or condition(s) directly leading to death, 2) any morbid conditions leading to the condition(s) that directly caused death, and 3) any other significant conditions contributing to the death, but not related to the disease(s) or condition(s) causing it. The diagnoses were coded using International Classification Disease (ICD-10) codes. If a minimum of 2 reviewers agreed, a cause of death was ascribed. If there was no agreement between the reviewers, a consensus meeting was held to reach an agreement where possible. If no agreement could be reached, the cause of death was recorded as unknown.

***Assessment of causality of (S)AEs***

The investigators used their clinical judgement to determine the relationship between the vaccine (or vaccination in case of co-administration of multiple vaccines) and the occurrence of each (S)AE. Alternative causes (natural history of the underlying diseases, concomitant therapy, other risk factors and the temporal relationship of the event to vaccination) had to be considered and investigated. The investigator also had to consult the investigator brochure. An assessment of causality had to be made for every event prior to submission of the SAE report. The investigators could change their opinion and amend the SAE information based on follow-up information. The following question was used to assess causality:

Is there a reasonable possibility that the (S)AE may have been caused by the study vaccine?

- No: The AE is not causally related to administration of the study vaccine(s). There are other, more likely causes and administration of the study vaccine(s) is not suspected to have contributed to the AE.
- Yes: There is a reasonable possibility that the vaccine(s) contributed to the (S)AE.

Supplemental measurements or evaluations could be requested to elucidate as fully as possible the nature and/or causality of the (S)AE. In case of a death during the study or extension period, a copy of any available post-mortem findings, including histopathology, and any verbal autopsy assessment had to be provided.

***Expert review of meningitis***

Two independent external experts (selected based on their expertise in infectious and pediatric diseases) reviewed all SAEs with central nervous system (CNS) infection/inflammation. The experts received the following data: background on the RTS,S/AS01 vaccine and the meningitis signal in the study; study protocol and literature publications related to this study; a table summarizing relevant safety information for all cases (treatment group assignment was not made available to the experts); the Council for International Organizations of Medical Sciences form of all CNS infection/inflammation cases with the case narrative. The cases were categorized as: “confirmed meningitis”, “no meningitis”, or “undetermined”.

***Expert review of cerebral malaria***

Two independent external experts (selected based on their background in pediatrics, infectious diseases and/or neurology, and their expertise in African settings) reviewed all hospitalized severe malaria cases with >0 *P. falciparum* parasites/µL and at least one neurological marker of severe disease (BCS ≤2, 2 or more seizures, or prostration) in order to identify and/or clinically confirm cerebral malaria (CM) cases. The experts received the following data: background on the RTS,S/AS01 vaccine; study protocol; literature publications related to this study; a table summarizing the criteria used for the selection of cases to be reviewed by the experts (treatment group assignment was not made available to the experts); an Excel table, including the main laboratory findings, of all the cases of severe malaria identified by the search criteria; the case narrative for all identified cases. The cases were categorized as “confirmed CM”, “uncertain CM”, or “not CM”. Of note, in addition to the working case definition, the experts were also asked/advised to employ their clinical judgment.

**References**

1. Swysen C, Vekemans J, Bruls M, Oyakhirome S, Drakeley C, Kremsner P, Greenwood B, Ofori-Anyinam O, Okech B, Villafana T, et al. Development of standardized laboratory methods and quality processes for a phase III study of the RTS, S/AS01 candidate malaria vaccine. Malar. J. 2011;doi: 10:223. 10.1186/1475-2875-10-223
